# Detection of small RNAs in *Bordetella pertussis *and identification of a novel repeated genetic element

**DOI:** 10.1186/1471-2164-12-207

**Published:** 2011-04-27

**Authors:** David Hot, Stéphanie Slupek, Bérénice Wulbrecht, Anthony D'Hondt, Christine Hubans, Rudy Antoine, Camille Locht, Yves Lemoine

**Affiliations:** 1 Center for Infection and Immunity of Lille (CIIL), Institut Pasteur de Lille, Lille, France; 2 U1019, Institut National de la Santé et de la Recherche Médicale, Lille, France; 3 UMR8204, Centre National de la Recherche Scientifique, Lille, France; 4 Univ Lille Nord de France, Lille, France; 5 IFR142, Molecular and Cellular Medicine, Lille, France; 6 Genoscreen, Campus Institut Pasteur de Lille, Lille, France

## Abstract

**Background:**

Small bacterial RNAs (sRNAs) have been shown to participate in the regulation of gene expression and have been identified in numerous prokaryotic species. Some of them are involved in the regulation of virulence in pathogenic bacteria. So far, little is known about sRNAs in *Bordetella*, and only very few sRNAs have been identified in the genome of *Bordetella pertussis*, the causative agent of whooping cough.

**Results:**

An *in silico *approach was used to predict sRNAs genes in intergenic regions of the *B. pertussis *genome. The genome sequences of *B. pertussis, Bordetella parapertussis, Bordetella bronchiseptica *and *Bordetella avium *were compared using a Blast, and significant hits were analyzed using RNAz. Twenty-three candidate regions were obtained, including regions encoding the already documented 6S RNA, and the GCVT and FMN riboswitches. The existence of sRNAs was verified by Northern blot analyses, and transcripts were detected for 13 out of the 20 additional candidates. These new sRNAs were named *Bordetella pertussis *RNAs, *bpr*. The expression of 4 of them differed between the early, exponential and late growth phases, and one of them, *bprJ2*, was found to be under the control of BvgA/BvgS two-component regulatory system of *Bordetella *virulence. A phylogenetic study of the *bprJ *sequence revealed a novel, so far undocumented repeat of ~90 bp, found in numerous copies in the *Bordetella *genomes and in that of other Betaproteobacteria. This repeat exhibits certain features of mobile elements.

**Conclusion:**

We shown here that *B. pertussis*, like other pathogens, expresses sRNAs, and that the expression of one of them is controlled by the BvgA/BvgS system, similarly to most virulence genes, suggesting that it is involved in virulence of *B. pertussis*.

## Background

Small bacterial RNAs (sRNAs) have recently been shown to participate in the regulation of gene expression, and have been identified in numerous prokaryotic species [1-4]. They act mainly by antisense base pairing with their target mRNAs, often within a complex comprising the Sm-like RNA chaperone Hfq [5-7] or by direct binding to proteins resulting in the modulation of their activity [8,9]. Some sRNAs are involved in the regulation of virulence in several pathogenic bacteria [10-16]. These sRNAs function either directly on virulence genes or on their regulators. They act in parallel with protein regulatory systems in order to fine-tune the expression of virulence genes. For example, the *Staphylococcus aureus *RNAIII regulatory RNA is the effector molecule of the quorum sensing system *arg*, composed of a two-component system (ArgA/C) sensing a small autoinducing peptide, which binds and activates ArgC. This autoinducing peptide is processed from the propeptide (ArgD) by the peptidase (ArgB) [17].

In this work we focused on the pathogenic bacterium *Bordetella pertussis*, the causative agent of whooping cough, which remains an important global health problem, with up to 300,000 annual deaths and approximately 45 million cases each year [18,19]. Most deaths occur in young, unvaccinated children, but the incidence is also increasing in vaccinated populations, with regular epidemic outbreaks since the 1990 s [20-22]. Several causes have been attributed to this resurgence, including increased awareness and improved diagnostics of the disease, waning of vaccine-induced immunity and pathogen adaptation [19,22].

A large proportion of *Bordetella *genes undergo a change in their expression during the infectious cycle [23-25] some of which correspond to phenotypic modulation under the control of a two-component system named BvgA/BvgS (reviewed in [26]). In this system BvgS acts as a sensor anchored in the inner membrane and BvgA as the transcriptional activator [27,28]. So far, little is known about sRNAs in *Bordetella *and their potential role in virulence or adaptation. Only three *Bordetella *sRNAs have been identified, the 6S RNA [29], the tmRNA [30] and a sRNA discovered by serendipity and lying in the opposite direction of the *bvgA/S *mRNA 5' untranslated region (UTR) [31,32]. The aim of this study was to scan the *B. pertussis *genome in order to identify potential sRNAs and to investigate whether some of them might be related to bacterial virulence. We used a general bioinformatics approach [33] and predicted 20 locations putatively bearing sRNA genes in intergenic regions (IGR) of the *B. pertussis *genome. Transcription was confirmed by Northern blot analyses for 13 of these locations. We further studied the expression of these new sRNAs under phenotypic modulation and showed that one of them is under the control of the BvgA/BvgS system, suggesting its role in virulence.

## Methods

### Bacterial strains and growth conditions

The genome sequences of *B. pertussis *TohamaI, *Bordetella parapertussis *12822, *Bordetella bronchesipteca *RB50 [34] and *Bordetella avium *N197 [35] were used in the computational analyses.

*B. pertussis *BPSM, a streptomycin-resistant derivative of TohamaI [36], and BPLOW, an avirulent *bvgAS *deletion mutant [37], were grown as previously described [24] at 37°C in modified Stainer-Scholte medium, containing 100 μg/ml streptomycin (Sigma Chemicals). Cultures were stopped either at early (OD_600 nm _~ 0.9), exponential (OD_600 nm _~ 1.8) or stationary phase (OD_600 nm _~ 3.8) by adding 2 ml of 5:95 (v:v) phenol/ethanol to 8 ml of culture medium. After centrifugation for 8 min. at 2800 × *g *the pellets were stored at -80°C until further use.

For phenotypic modulation a 630-ml culture was grown in the same conditions until an OD_600 nm _of approximately 0.7. The culture was then split into three, and phenotypic modulation was induced by adding 40 ml of a pre-warmed 125 mM nicotinic acid (20 mM final concentration) or 40 ml of pre-warmed 300 mM MgSO_4 _(48 mM final concentration) in the two first sub-cultures. The third sub-culture served as control and received 40 ml of sterile water pre-warmed at 37°C. Culturing was then continued at 37°C, and samples were taken for RNA extraction, just before induction (t = 0) and at t = 1 min., 10 min., 1 h, 2 h, 6 h, 10 h and 30 h post-induction.

### *In silico *search of sRNAs

The bacterial genome sequences and annotation files were obtained from the NCBI databases [38] and from the Sanger Institute [39]. Gene coordinates were used to extract IGRs using a parser in Perl. Homologous regions of these IGR sequences were searched in different bacterial genomes using local BLAST. Hits presenting more than 60% sequence identity over at least 70 nucleotides were analyzed further. A multiple alignment of these homologous regions was then obtained using ClustalW. These alignments were used to evaluate secondary structure conservation and thermodynamic stability using the software "RNAz" developed by Washietl *et al*. [33,40].

### RNA extraction and Northern blot analyses

The bacterial pellets were resuspended in 200 μl of 1 mg/ml Lysozyme (Sigma Aldrisch). Total RNA was then extracted using the TRI Reagent kit (Ambion), following the recommendations of the supplier. The RNA quality was checked using Bioanalyzer 2100 (Agilent Technologies) before denaturation and electrophoresis (10 μg per lane) on a 10% acrylamide:bis-acrylamide (37.5:1) denaturing gel in 0.5 × TBE buffer in the presence of 8 M urea. The RNA was then transferred onto BrightStar Plus (Ambion) nylon membranes and UV-crosslinked. Biotinylated oligonucleotides (see Additional file 1, table S1), designed using FastPCR [41], were then used for hybridization in Northern Max buffer (Ambion), and the blots were developed by chemiluminescent detection using the BrightStar Biodetect kit (Ambion).

### Local DNA alignment

Homology searches were performed with the program YASS [42] in local using the default parameters.

For the similarity search of the *bprJ1 *sequence, the program YASS was used on the web server [43] using the default parameters, except for the 'Hit criterion strategy', which was set on 'double hit'. The sequence of the IGR containing *bprJ1 *(between positions 3605200 and 3605405) was searched for homology in the *B. pertussis *genome. The homologous regions were then aligned using ClustalW, and the sequence closest to the consensus sequence (*i.e*. the sequence from position 2208359 to 2208448) was used as a query for the search in all bacterial genome sequences.

For the global sequence similarity search, the *B. pertussis *IGR sequences longer than 140 nucleotides and not containing tRNA or rRNA genes were compared using YASS with all complete bacterial genome sequences available in GenBank, except those of the *Bordetella *genus. Hits with a score >50 and with >70% identity were considered further.

## Results and discussion

### In silico prediction of sRNAs and validation by Northern blot analysis

Except for the 6S RNA [29], the tmRNA [30] and a small RNA transcribed in antisense to the *bvgA *mRNA [31,32], no sRNA has been identified so far in *Bordetella*. We used the 'RNAz' algorithm [33], which is based on a search for structure conservation between closely related bacterial genomes. Predictions using different combinations of betaproteobacteria genomes resulted in various numbers of candidates. We choose to work on a prediction obtained comparing genomes of *B. pertussis *Tohama I, *B. bronchiseprica *RB50, *B. parapertussis *12822 and *B. avium *197N. This search resulted in the prediction of 23 sequences for potential sRNAs in the *B. pertussis *genome (see Additional file 1, table S2 for the detailed list of predictions). The same search strategy using only *B. pertussis *Tohama I, *B. bronchiseprica *RB50 and *B. parapertussis *12822 resulted in 657 predictions. Among the 23 predicted sequences, the position of the 6S RNA gene was correctly designated (predicted coordinates from 3246822 to 3246972, real coordinates from 3246812 to 3246993), as well as the position of 2 riboswitches already described in the Rfam database [44]. The first, GCVT, is selectively triggered by glycine [45] and controls the translation of the aminomethyltransferase mRNA (BP0195). The second riboswitch belongs to the flavin mononucleotide (FMN) class [46] and regulates the translation of *ribB *(BP0471).

### Validation by Northern blot analysis

**Figure 1:**
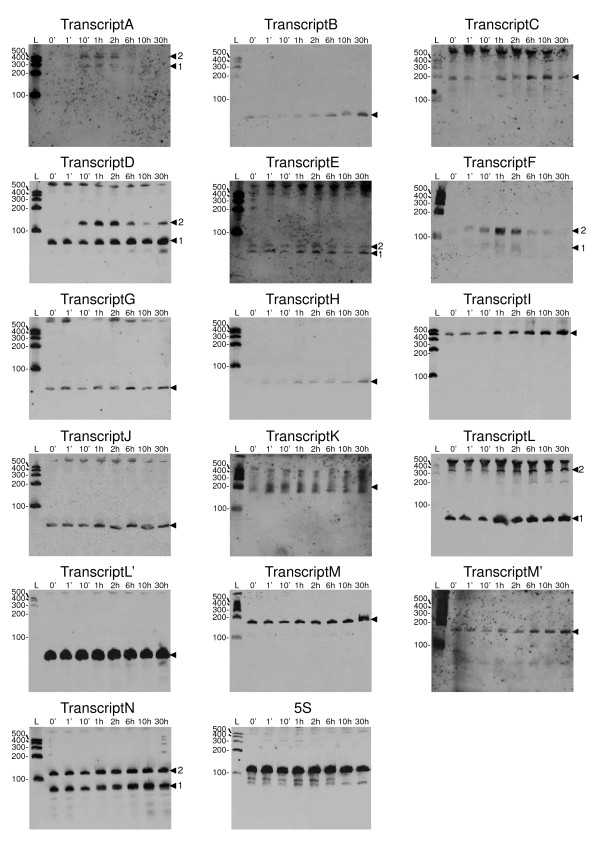
**Detection of transcripts by Northern blot analyses**. Total RNA was extracted from *B. pertussis *Tohama I at indicated times after sub-culturing, and 10 μg total RNA per lane was used for Northern blot analyses. The detected transcripts are indicated by arrow heads (and numbers in case of multiple bands). L, RNA size ladder. RNA sizes are indicated in nucleotides in the left margins of each panel. The detection of 5S rRNA was used as loading control.

All 23 predicted regions, except the ones of the GCVT riboswitch, the FMN riboswitch and the 6S RNA, were tested for transcription of small-size RNA by Northern blot analyses. Biotinylated oligonucleotide probes were designed for both strands of the 20 predicted regions (see probe sequences in Additional file 1, table S1) and used to test the presence and orientation of short transcripts in Northern blot analyses. As a positive control, an additional probe was designed that corresponds to the sRNA characterized by Scarlato *et al*. [31,32]. When total RNA extracted from *B. pertussis *cultures stopped at an ODs_600 nm _of 0.9, 1.8 or 3.6 was analyzed, 13 positions in addition to the positive control showed evidence of transcription (see Additional file 1, figure S1). The transcription profile of these 14 genomic regions was further analyzed for the kinetics of expression over 30 h of culture [from OD_600 nm _~0.7 to ~7.9] (Figure 1). All the bands clearly detected in the Northern blots in the validation experiments (Figure 1 and Additional file 1, figure S1) and in the phenotypic modulation experiments (see below) were considered as transcripts. Each detected transcript was labeled with a capital letter. A number was added after the letter to discriminate transcripts in case of multiple bands (*e.g*. TranscriptA1, TranscriptA2). Locations for which transcription was detected on both strands were distinguished with an apostrophe, *e.g*. TranscriptM, TranscriptM' (Figure 1 and Table 1).

**Table 1:** Summary of identified sRNA features

*Transcript name*	*Gene name*	*Predicted 5' coordinate^a^*	*Predicted 3' coordinate^a^*	*Predicted length^b^*	*Approx. obs. length^c^*	*sRNA orientation^d^*	*IGR length^e^*	*5' gene name and orientation^f^*	*3' gene name and orientation^f^*
TranscriptA1	***bprA1***	488504	488654	150	250	<	522	BP0475 <	BP0477 >
TranscriptA2	***bprA2***	488504	488654	150	300	<	522	BP0475 <	BP0477 >
TranscriptB	***bprB***	1494130	1494207	77	80	>	726	BP1418 <	BP1419 >
TranscriptC	***bprC***	1968374	1968529	155	190	>	425	BP1878 <	BP1879 >
TranscriptD1	***bprD1***	2624007	2624157	150	90	<	783	BP2479 <	BP2480 >
TranscriptD2	***bprD2***	2624007	2624157	150	110	<	783	BP2479 <	BP2480 >
TranscriptE1	***bprE1***	2699394	2699487	93	70	>	239	BP2546 <	BP2547 >
TranscriptE2	***bprE2***	2699394	2699487	93	80	>	239	BP2546 <	BP2547 >
TranscriptF1	***bprF1***	3099570	3099720	150	80	<	256	BP2908 <	BP2909 <
TranscriptF2	***bprF2***	3099570	3099720	150	150	<	256	BP2908 <	BP2909 <
TranscriptG	***bprG***	3173584	3173734	150	70	<	305	BP2982 >	BP2983 >
TranscriptH	***bprH***	3178090	3178331	241	70	>	318	BP2984 >	BP2985 >
TranscriptI	***bprI***	3263729	3263815	86	450	<	277	BP3061 >	BP3062 >
TranscriptJ	***bprJ***	3605317	3605397	80	70	>	206	BP3395 <	BP3396 <
TranscriptK	***bprK***	3619230	3619365	135	200	<	316	BP3410 <	BP3411 >
TranscriptL1	***bprL1***	3811548	3811699	151	80	<	336	BP3594 <	BP3595 >
TranscriptL2	***bprL2***	3811548	3811699	151	350	<	336	BP3594 <	BP3595 >
TranscriptL'	***bprL'***	3811548	3811699	151	80	>	336	BP3594 <	BP3595 >
TranscriptM	***bprM***	3896371	3896457	86	190	<	281	BP3686 >	BP3687 >
TranscriptM'	***bprM'***	3896371	3896457	86	190	>	281	BP3686 >	BP3687 >
TranscriptN1	***bprN1***	3956837	3956934	97	80	<	393	BP3747 >	BP3748 <
TranscriptN2	***bprN2***	3956837	3956934	97	110	<	393	BP3747 >	BP3748 <

^a^Genomic coordinates as predicted by RNAz.

^b^In nucleotides.

^c^sRNA length, in nucleotides, determined from apparent sizes on Northern blots.

^d^Genomic orientation of sRNA transcripts. '>' = on positive strand: the strand given in the GenBank genome database (NC_002929); '<' = on negative strand: the complementary strand.

^e^Length, in nucleotides, of the IGRs containing sRNA genes (according to the annotation).

^f^Genomic orientation of flanking mRNAs. '>' = on positive strand: the strand given in the GenBank genome database (NC_002929); '<' = on negative strand: the complementary strand.

The analysis of five genomic positions resulted in the detection of 2 bands (transcripts A1 and A2, D1 and D3, E1 and E2, L1 and L2, N1 and N2), indicating the presence of two overlapping transcripts of different lengths, as has been seen for several sRNAs of *Pseudomonas aeroginosa*[47] and for *Escherichia coli *IstR1 and IstR2, which have each a specific function and are generated from separate promoters but share a common 5' end [48]. Alternatively, these bands could be the result of a post-transcriptional processing of a single RNA transcript [47].

**Figure 2:**
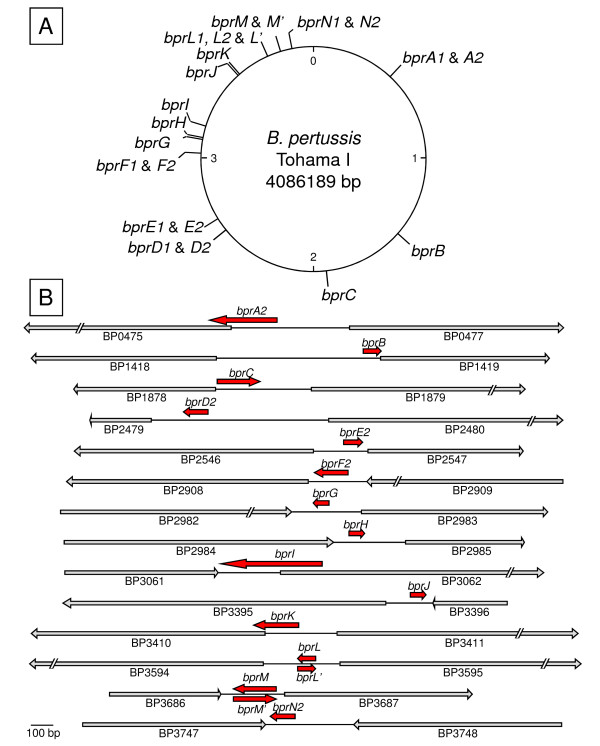
**Chromosomal position and genomic context of the *bpr *loci of *B. pertussis***. The positions of the 14 *bpr *loci are shown on the chromosomal map of the *B. pertussis *genome. The numbers give an approximate chromosome graduation in Mbp (A). The positions and orientations of the *bpr *loci in the *B. pertussis *genome are depicted by the red arrows (B). Grey arrows represent the ORF of the *bpr*-adjacent genes and their orientations according to the GenBank annotation. The estimated positions of sRNAs were determined considering the positions of the biotynilated probes (see Additional file 1, table S2) and the apparent lengths of the transcripts on the Northern blots. In case of multi-band detection only the longest transcript is represented.

Some transcripts were only produced during the exponential phase (transcripts A1, A2 and F). Transcript D1 was expressed at higher levels during exponential phase compared to the early and stationary phases, whereas Transcript D2 did not show any significant modification of expression during growth. This observation rules out the possibility that Transcript D1 is the precursor of Transcript D2, and argues that the two transcripts are independent and under the control of different factors. In contrast, Transcripts A1, A2 and Transcripts F1, F2, are expressed at similar levels during the different growth phases. They may therefore each be produced from a unique RNA and subsequently processed into a shorter version. Finally, two regions gave rise to a transcript from both strands. Transcripts L1 and L' are transcribed respectively from the lagging and leading strand template of predicted region 3811548-3811699. Transcripts M and M' are transcribed respectively from the lagging and leading strand template of predicted region 3896371-3896457. In both cases, the two transcripts had the same apparent size (~80 nucleotides for transcripts L1 and L' and ~190 nucleotides for transcripts M and M'). For these two pairs of transcripts, the biotinylated probes used to detect transcription on both strands were designed approximately at the same coordinates, indicating that the observed transcripts are complimentary to each other for most of their lengths (Figures 1 and 2B).

Most regulatory sRNAs are synthesized as discrete transcripts under the control of dedicated promoter and terminator sequences. However, some bacterial regulatory elements correspond to sequences at the 5' or 3' UTRs of mRNAs that can adopt different conformations in response to environmental signals. This is the case for autoregulatory motifs and riboswitches [49]. Some of these regulatory elements can, under specific conditions, cause transcriptional arrest (reviewed in [50]). In order to test whether some of the transcripts detected in this study are abortive, processed or degraded forms of the 5'UTR or 3'UTR of their neighboring genes, a control RT-PCR was carried out on transcripts which were in close vicinity of and in the same orientation as their neighboring 5' or 3' ORF (see Additional file 2, figure S2 and table S3). All detected transcripts were found to result from independent transcription, except for Transcript B, which was linked to the transcription of the downstream gene *rpsB *(BP1419), encoding the 30S ribosomal protein S2. The function of S2 is still uncertain. It may potentially act as a bridge between the 16S RNA and ribosomal protein S1 [51]. The ORF of *rpsB *genes have recently been shown to be preceded by a conserved specific motif in the 5'UTR, which could be a cis-regulatory element binding to the S2 protein for an autoregulatory control of its own synthesis [52]. The existence of Transcript B suggests that in *B. pertussis *the 5'UTR of *rpsB *contains a cis-regulatory element inhibiting the full-length transcription of the mRNA, as it has been demonstrated for other ribosomal protein genes [53,54].

Finally, the coding potential of all transcripts was evaluated by a search for potential coding sequences in their genomic regions using the program 'ORF Finder' of the NCBI [55] (data not shown). As the *B. pertussis *genome appears to be under-annotated when the EasyGene prediction is compared to the RefSeq annotation [56], we also looked for predicted coding sequences or neighboring genes with alternative start codons in these genomic regions, using the prokaryotic gene-finder algorithm EasyGene. No coding sequences were predicted in the transcribed regions, even for the longest ones.

In view of these observations we conclude that all detected transcripts are genuine sRNAs and were therefore named *Bordetella pertussis *RNAs (BprA1, A2, B, C, ...). The *rpsB *gene probably contains in its 5'UTR an autoregulatory element, and BprB most likely results from the inhibition of transcription of the *rpsB *mRNA. The validated sRNA genes are scattered throughout the *B. pertussis *chromosome (Figure 2A). The location of these sRNA genes relatively to their neighboring genes was determined by the biotinylated probe coordinates and the apparent sizes on the Northern blots (Figure 2B).

### Features of the B. pertussis sRNAs

Specific and general features of the *B. pertussis *sRNAs were further analyzed and compared with those of other bacterial sRNAs. The genes of the *B. pertussis *sRNAs are located in IGR of various lengths, comprised between 206 bp and 783 bp. They have no apparent preference for lagging or leading strand templates, but they are preferentially located on one of the two replichores (79%) (See Figure 2A). These characteristics are similar to those of the *E. coli *sRNA genes [57].

Some of the *B. pertussis *sRNA genes (BprC, I and N) are very close to or overlap adjacent ORFs in the opposite orientation and thus are likely to act on their mRNA, whereas others are more distant from the adjacent ORFs and/or in the same orientation (See Figure 2B). The potential mRNA targets of all sRNAs were predicted using TargetRNA algorithm [58]. The predicted gene targets were compared to documented targets of sRNAs from other bacteria. However, none of the predicted *B. pertussis *mRNA targets was orthologous to a sRNA target from another bacterium (data not shown). Similarly, orthologous genes of the *B. pertussis *sRNA-flanking genes were searched in the Rfam and Noncode databases, as some sRNAs are cis-encoded antisense or cis-acting elements. The only product mentioned in these databases is the ribonuclease E gene (ortholog to BP0475), which flanks *bprA *and is in the same orientation. BprA might therefore be the 5'UTR cis-acting element of the ribonuclease E gene (*rne *5'UTR), which codes for a key enzyme in the mRNA degradation pathway, including its own. This *rne *5'UTR element acts as a sensor of the cellular RNase E concentration and allows for auto-regulation of its mRNA degradation [59].

### Phenotypic modulation of B. pertussis sRNAs

**Figure 3:**
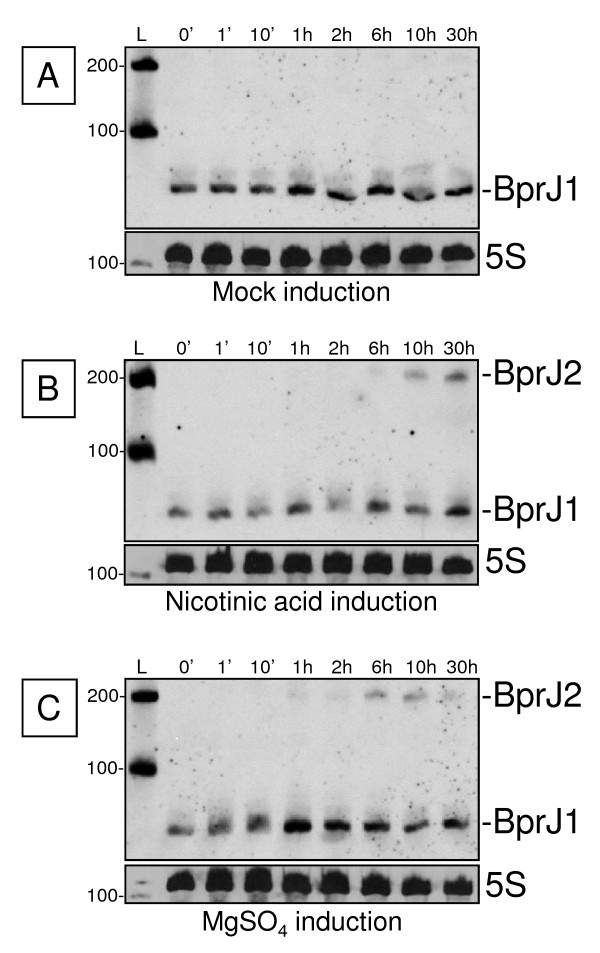
**Phenotypic modulation of *bprJ *expression**. RNA was extracted at the indicated times after sub-culturing and induction by H_2_O (panel A: mock-induction), nicotinic acid (panel B) or MgSO_4 _(panel C). BprJ1 and BprJ2 transcript positions are indicated. L, RNA size ladder. RNA sizes are indicated in nucleotides in the left margins of each panel. The detection of 5S rRNA was used as loading control.

To investigate whether some of the new sRNAs genes may potentially be under the control of the two-component system BvgA/S and therefore potentially be involved in *Bordetella *virulence regulation, phenotypic modulation was induced by adding nicotinic acid or MgSO_4 _in the growth medium in order to switch the bacteria from the virulent (Bvg^+ ^phase) to the non-virulent phase (Bvg^-^ phase) [60,61]. The modulation of expression was monitored by Northern blot analyses for up to 30 h after induction (data not shown) and the expression profiles were compared to those of the non-induced culture. One sRNA, BprJ, showed a change in its expression compared to the control culture. The 80-nucleotides band detected in the non-modulated culture did not change after phenotypic modulation, but a second band of approximately 200 nucleotides appeared 6 h and 10 h after induction of phenotypic modulation by MgSO_4 _and nicotinic acid, respectively (Figure 3). This new band is indicative of longer transcript overlapping the 80-nucleotide transcript and appears to be specific for the non-virulent phase (Bvg^-^ phase). According to the nomenclature adopted above, this second transcript was named BprJ2 to distinguish it from the 80-nucleotides transcript, which was named BprJ1.

**Figure 4:**
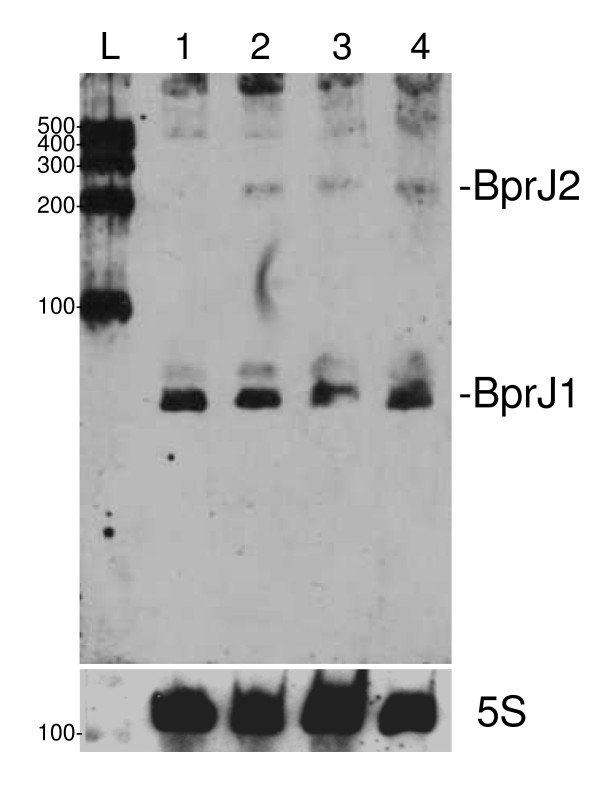
**Expression of *bprJ2 *in BPLOW**. RNA was extracted at t = 10 h from a non-modulated *B. pertussis *culture (lane 1), from a MgSO_4 _modulated culture (lame 2), from a nicotinic acid modulated culture (lane 3) and from a BPLOW culture (lane 4). BprJ1 and BprJ2 transcript positions are indicated. L, RNA size ladder. RNA sizes are indicated in nucleotides in the left margins of each panel. The detection of 5S rRNA was used as loading control.

To confirm that this new transcript is under the control of the BvgA/BvgS system, its expression was monitored by Northern blot analysis in the avirulent BPLOW mutant in which part of the *bvgA*-*bvgS *operon had been deleted [37]. BprJ2 was also detected in the BPLOW RNA (Figure 4), confirming its *vrg *(virulence repressed gene) feature. The other *vrg*s of *B. pertussis *mainly encode cell-envelope, small-molecule degradation or hypothetical proteins [23,24] and include the previously described but uncharacterized *vrg6, vrg18, vrg24 *and *vrg73 *genes [62].

### Identification of a novel Betaproteobacteria repeated element

A local DNA sequence analysis using YASS showed that the *bprJ1 *and *bprJ2 *genomic region contains a sequence, from coordinates 3605317 to 3605401, highly repeated in the *Bordetella *(see Additional file 1, list S1) and in several other Beta-proteobacteria genomes (Table 2). We named it therefore Beta-proteobacterial repeat element (BRE). This repeated element is found as a full-length sequence of ~90 bp or as partial but conserved sequence (between ~60 and ~40 bp) with a maximum number of 29 repeats (with scores > 50 and > 70% identity) in the genome of *Polaromonas sp*. JS666 (see Additional file 1, table S4). All identified repeated elements were located in chromosomal DNA, usually in IGRs, although some overlap ORFs at their 5' or 3' ends by a few bases. Surprisingly, some of the repeats are located entirely within coding sequences.

**Table 2:** Number of BRE detected in different bacterial strains

*Nbr. of repeats*	*Strain*	*Familly*	*Acc.number*
8	Bordetella avium 197N	Alcaligenaceae	NC_001064
22	Bordetella bronchiseptica RB50	Alcaligenaceae	NC_002927
10	Bordetella parapertussis 18822	Alcaligenaceae	NC_002928
8	Bordetella pertussis Tohama I	Alcaligenaceae	NC_002929
1	Azoarcus sp. EbN1	Rhodocyclaceae	NC_006513.1
4	Rhodoferax ferrireducens T118	Comamonadaceae	NC_007908.1
29	Polaromonas sp. JS666	Comamonadaceae	NC_007948.1
4	Acidovorax avenae subsp. citrulli AAC00-1	Comamonadaceae	NC_008752.1
4	Polaromonas naphthalenivorans CJ2	Comamonadaceae	NC_008781.1
1	Acidovorax sp. JS42	Comamonadaceae	NC_008782.1
1	Verminephrobacter eiseniae EF01-2	Comamonadaceae	NC_008786.1
13	Delftia acidovorans SPH-1	Comamonadaceae	NC_010002.1

**Figure 5:**
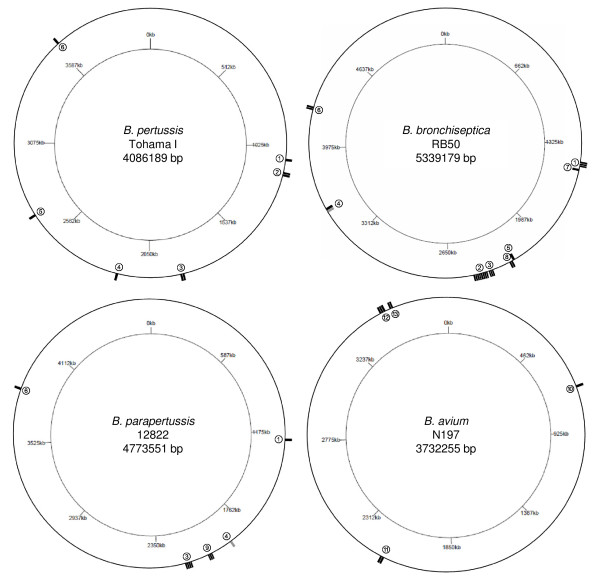
**Distribution of BRE throughout the genomes of *B. pertussis*, *B. bronchiseptica*, *B. parapertussis *and *B. avium***. Repeats in the positive orientation (on the strand given in the GenBank genome database) are represented by a tick on the outside of the outer circles, and those in the negative orientation (on the complementary strand) are represented by a tick on the inside of the outer circles. The ticks in black are full-length repeats and ticks in grey are partial-length repeats. The number of ticks at each locus corresponds to the numbers of repeats. The inner circles give a chromosome graduation in kbp. Numbers on the outer circle indicate the locus number as defined in table 4.

Some IGRs and ORFs contain more than one BRE (a maximum of 8 repeats was observed in *B. bronchiseptica *between BB2301 (*sphB3*) and BB3202) organized in tandem with no or only a few nucleotides between each repeat. The complete and partial BRE sequences are scattered around the chromosomes of *B. pertussis, B. bronchiseptica, B. parapertussis *and *B. avium *(Figure 5). An analysis of the *Bordetella *BRE sequences revealed 44 full-length repeats, ranging from 75 to 91 bp in length, and 1 partial repeat of 50 bp. They exhibit an average GC-content of 69.0% (ranging from 63.7% to 78.9%), which is higher than the average GC-content of IGR sequences (62.9% for IGRs ≥ 10 bp) and slightly higher than the average GC-content of the complete genome sequences (67.7%). The orientations of the repeats, inferred by the transcriptional orientation of *bprJ1 *and *bprJ2*, are not co-orientated with the direction of the replication fork.

The genetic context, *i.e*. the nature and orientation of the BRE flanking genes, is only moderately conserved between *B. pertussis, B. bronchiseptica *and *B. parapertussis *(see table 3). For instance, only 4 (#1, 3, 4 and 6) out of the 9 loci from *B. pertussis, B. bronchiseptica *or *B. parapertussis *show conservation of the genetic context in the 3 genomes (table 3). None of the genetic contexts is conserved in the *B. avium *genome (loci #10 to 13).

**Table 3:** BRE *loci *genetic context in *Bordetella *genomes

	*B. pertussis genetic contexte*					*B. bronchiseptica genetic contexte*					*B. pararpertussis genetic contexte*					*B. avium genetic contexte*				
Locus #	Rpts. nbr.^a^	5' gene	Std.^b^	3' gene	Std.^b^	Rpts. nbr.^a^	5' gene	Std.^b^	3' gene	Std.^b^	Rpts. nbr.^a^	5' gene	Std.^b^	3' gene	Std.^b^	Rpts. nbr.^a^	5' gene	Std.^b^	3' gene	Std.b
1	1	BP1052	>	BP1053	>	3	BB1365	>	BB1366	>	1	BPP1149	>	BPP1150	>	-	-	-	-	-
2	2	BP1110	>	BP1111	>	8	BB2301	>	BB2302	>	-	-	-	-	-	-	-	-	-	-
3	2	BP1793	>	BP1794	<	3	BB2270	>	BB2271	<	4	BPP2022	>	BPP2023	<	-	-	-	-	-
4	1	BP2085	>	BP2086	>	2	BB3338	<	BB3339	<	1	BPP1769	>	BPP1770	>	-	-	-	-	-
5	1	BP2549	>	BP2550	<	1	BB2072	>	BB2073	<	-	-	-	-	-	-	-	-	-	-
6	1	BP3395	<	BP3396	<	2	BB3983	<	BB3984	<	1	BPP3548	<	BPP3549	<	-	-	-	-	-
7	-	-	-	-	-	1	BB1410	>	BB1411	>	-	-	-	-	-	-	-	-	-	-
8	-	-	-	-	-	2	BB2091	>	BB2092	>	-	-	-	-	-	-	-	-	-	-
9	-	-	-	-	-	-	-	-	-	-	3	BPP1903	>	BPP1904	>	-	-	-	-	-
10	-	-	-	-	-	-	-	-	-	-	-	-	-	-	-	1	BAV0696	>	BAV0697	>
11	-	-	-	-	-	-	-	-	-	-	-	-	-	-	-	2	BAV2006	>	BAV2007	>
12	-	-	-	-	-	-	-	-	-	-	-	-	-	-	-	3	BAV3179	>	BAV3180	<
13	-	-	-	-	-	-	-	-	-	-	-	-	-	-	-	2	BAV3208	>	BAV3209	<

^a^'Rpts. nbr.' = Number of Repeats. Number of repeated BRE elements at the specific locus.

^b^'Std.' = Strand. Genomic orientation of flanking mRNAs. '>' = on positive strand: the strand given in the GenBank genome database; '<' = on negative strand: the complementary strand.

**Figure 6:**
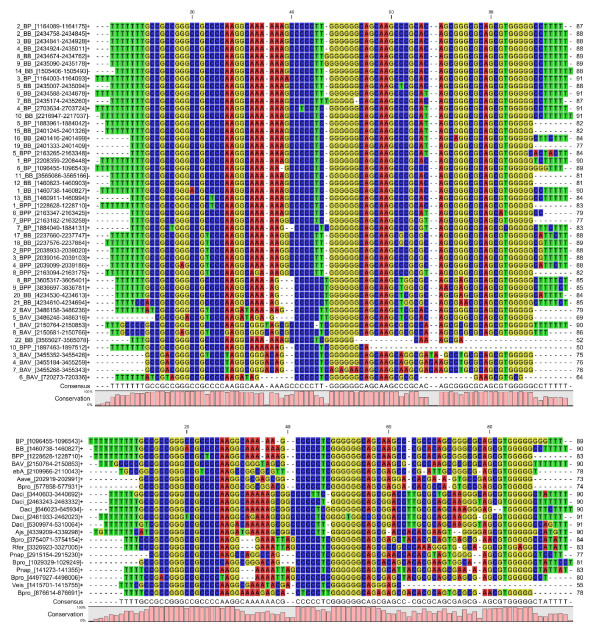
**Multiple-alignment of *Bordetella *and betaproteobacteria BREs**. The sequences were aligned using CLC Genomics Workbench 4. The name code of the bacterial strain and the repeat coordinates on the genomes are indicated on the left. Indels are represented by dashes. Upper panel: the 45 BREs from the 4 genomes *B. pertussis*, *B. bronchiseptica*, *B. parapertussis*, *B. avium *were aligned to evaluate conservation within the *Bordetella *genus. Lower panel: The BREs with the smallest coordinate in each betaproteobacteria genome were aligned to evaluate conservation within the all classes.

For some loci the numbers of tandem repeats vary between the different genomes. For example, locus #3 contains 2 repeats in *B. pertussis *(between BP1793 and BP1794, *lexA*), 4 in *B. parapertussis *(between BPP2022 and BPP2023, *lexA*) and 3 in *B. bronchiseptica *(between BB2270 and BB2271, *lexA*) (table 3, Figure 5). Locus #2 is conserved only in *B. pertussis *and *B. bronchiseptica*. It has 2 repeats in *B. pertussis *(between BP1110, *sphB3*, and BP1111) and 8 in *B. bronchiseptica *(between BB2301, *sphB3*, and BB2302). Two out of 8 loci in *B. bronchiseptica *(loci #7 and 8) have no homologous locus in the other genomes, and 1 out of 5 loci in the *B. parapertussis *genome (locus 9) has no homologous locus in the other genomes. A multiple alignment analysis on the *Bordetella *repeats shows a high level of conservation within the genus, as well as more widely within the all Betaproteobacteria class (Figure 6).

Several small repeats have been documented over the years in other prokaryotic species, including ERICs in enterobacteria [63-65], NEMIS in *Neisseria *spp. [66-68], the BOX and RUP elements in *Streptococcus pneumoniae*[69-71], MIRUs in *Mycobacterium tuberculosis*[72] and the *bcr1 *element of *Bacillus cereus*[73,74]. These repeats often feature characteristics of mobile elements and have a role in mRNA stabilization, transcription termination or promoter activity. BRE, described for the first time in this study, needs further investigation before we can suggest any potential function. Its heterogeneous chromosomal distribution and the fact that it is sometimes found in multiple copies at a same locus might imply a mobility or a past-mobility property. A preliminary search for BRE in sequences of other strains of *B. pertussis, B. bronchiseptica *and *B. parapertussis *revealed the presence of variable numbers of the repeat at the same locus among different strains (data not shown). This qualifies BREs as Variable Number Tandem Repeats (VNTR). This feature was verified using a VNTR analysis program [75], and most of identified loci were correctly predicted as VNTR, except for locus 3 in *B. pertussis *and *B. bronchiseptica*, and for locus 4 in *B. bronchiseptica *(data not shown). Additional investigations are under progress to assess BRE as potential genotyping markers.

## Conclusion

*B. pertussis *and *B. parapertussis*, the second agent of whooping cough-like disease, have evolved relatively recently from a *B. bronchiseptica*-like ancestor, but these species have remained clonal, with a very limited number of *B. pertussis*-specific genes [76,77]. It is established that *B. pertussis *evolution and adaptation to humans occurred mainly through gene loss and recombination of the chromosome rather than through acquisition of new genes that would promote human infection [34]. It has also been suggested that certain genes are expressed differently in *B. pertussis *and in *B. bronchiseptica*, and that this difference might be responsible in part for infection in humans [78]. Such differences in expression may rely on classical base substitutions or indels in promoter regions of these genes but perhaps also on the specific action of unidentified regulatory molecules, such as sRNAs. In this study we have shown that *B. pertussis*, like other pathogens, expresses sRNAs and that the expression of one of them, BprJ2, is under the control of the BvgA/BvgS system. This sRNA and others, identified in this study or yet to be identified, could be important regulators for *B. pertussis *virulence acting either under the control of BvgA/BvgS as co-regulators or as independent virulence regulators.

## Competing interests

The authors declare that they have no competing interests

## Authors' contributions

DH carried out the planning, design, data analysis and direction of the project, and he drafted the manuscript. SS carried out experimental work. BW, AD and CH carried out bioinformatics and computational work. RA contributed to data analysis and interpretation and to the manuscript draft. CL and YL contributed to the planning and design of the project and to the manuscript. All authors read and approved the final manuscript.

## Supplementary Material

Additional file 1 **Additional figure S1 - Additional tables S1, S2, S4 - Additional list S1**. Figure S1: Preliminary Northern blot analysis. Detection of transcripts in early(E), exponential(Ex) and stationary(S) phases. Table S1: Biotin probe sequences used in northern blot analysis. Table S2: RNAz prediction details. Table S4: BRE details. List S1: Bordetelle BRE sequences and positions. click here for file

Additional file 2 **Additional figure S2 - Additional table S3**. Figure S2: Test for independent transcription between the putative sRNA positions and the upstream or downstream ORF and RT-PCR primers. Table S3: List of primers used in PCR described in figure S2. click here for file
