# Supplementary material for: Detection of small RNAs in Bordetella pertussis and identification of a novel repeated genetic element
Source: BMC Genomics. 2011 Apr 27;12:207. doi: 10.1186/1471-2164-12-207 (PMC3110155; doi:10.1186/1471-2164-12-207)
Supplement: Additional file 1 — Additional figure S1 - Additional tables S1, S2, S4 - Additional list S1. Figure S1: Preliminary Northern blot analysis. Detection of transcripts in early(E), exponential(Ex) and stationary(S) phases. Table S1: Biotin probe sequences used in northern blot analysis. Table S2: RNAz prediction details. Table S4: BRE details. List S1: Bordetelle BRE sequences and positions. [file 1471-2164-12-207-S1.PDF]

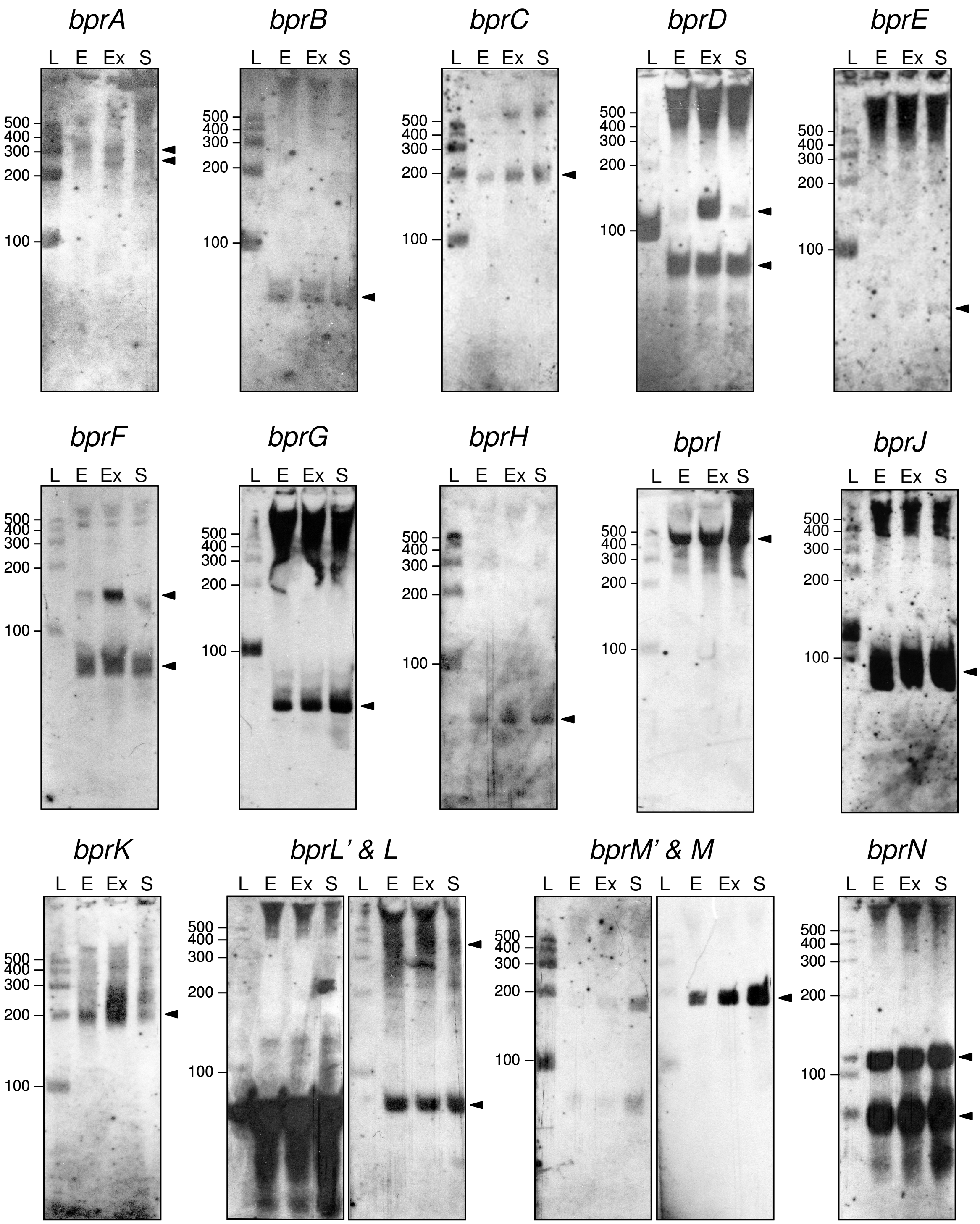
bprA
bprB
bprC
bprD
bprE
bprF
bprG
bprH
bprI
bprJ
bprK
bprL' & L
bprM' & M
bprN
L E Ex S
500
400
300
200
100

# Figure S1

Table S1: Biotin probe sequences

| Prediction name | Probe strand | Sequence | Length | Tm | GC-% |
|---|---|---|---|---|---|
| Pred_BP3686_BP3687 | F | cggagcttgctgttccgtcag | 21 | 61.7℃ | 61.9% |
| | R | gcccatctgacggaacagcaa | 21 | 61.0℃ | 57.1% |
| Pred_BP3410_BP3411 | F | agggtgaacgcatcgcggcca | 21 | 66.8℃ | 66.7% |
| | R | cggcgagacctttggccgcga | 21 | 67.4℃ | 71.4% |
| Pred_BP2546_BP2547 | F | cacgtataccctgagtgggca | 21 | 59.6℃ | 57.1% |
| | R | acaggggatggagcttgccca | 21 | 64.1℃ | 61.9% |
| Pred_BP2479_BP2480 | F | tgtctcctcacctgcatggga | 21 | 60.9℃ | 57.1% |
| | R | atggtgcagaatctcgtccca | 21 | 59.2℃ | 52.4% |
| Pred_BP0475_BP0477 | F | ggctgcggactgtagcgggca | 21 | 67.3℃ | 71.4% |
| | R | tcttgcccgctacagtccgca | 21 | 63.8℃ | 61.9% |
| Pred_BP2364_BP2365 | F | ggcaaacatcccattgtggca | 21 | 60.0℃ | 52.4% |
| | R | agacggagggcgtctgtgcca | 21 | 65.6℃ | 66.7% |
| Pred_BP1418_BP1419 | F | atccgatgcgggtgtagcgca | 21 | 64.0℃ | 61.9% |
| | R | ttgcgctacacccgcatcgga | 21 | 64.1℃ | 61.9% |
| Pred_BP0839_BP0840 | F | gaccatagccagtcctgccga | 21 | 62.0℃ | 61.9% |
| | R | tcggcaggactggctatggtc | 21 | 60.0℃ | 61.9% |
| Pred_BP3864_BP3865 | F | ccaacttcggctctccagcca | 21 | 62.7℃ | 61.9% |
| | R | gaagttggacaacatttcgga | 21 | 54.5℃ | 42.9% |
| Pred_BP0011_BP0013 | F | cagttcggatccagcgtagac | 21 | 58.4℃ | 57.1% |
| | R | caaacaaccggctagcgctcc | 21 | 62.3℃ | 61.9% |
| Pred_BP3529_BP3530 | F | atgcctggaagtacctgcgac | 21 | 60.3℃ | 57.1% |
| | R | cagagcagtattaggccttgg | 21 | 56.5℃ | 52.4% |
| Pred_BP2908_BP2909 | F | acttcagttaacacgcgtccc | 21 | 58.4℃ | 52.4% |
| | R | ccccgaaaaagccgccagcat | 21 | 64.3℃ | 61.9% |
| Pred_BP2982_BP2983 | F | attagtgccatacgagggggt | 21 | 58.7℃ | 52.4% |
| | R | tagtgtaccaccccctcgtat | 21 | 57.4℃ | 52.4% |
| Pred_BP3061_BP3062 | F | cttatgtgcagtggtggccgt | 21 | 61.0℃ | 57.1% |
| | R | ttatacggccaccactgcaca | 21 | 59.6℃ | 52.4% |
| Pred_BP3395_BP3396 | F | cccaaggcaaaagccccccctg | 21 | 65.0℃ | 66.7% |
| | R | caggggggcttttgccttggg | 21 | 65.0℃ | 66.7% |
| Pred_BP3752_BP3753 | F | cactgcaacattctcgacgca | 21 | 59.1℃ | 52.4% |
| | R | tgcgtcgagaatgttgcagtg | 21 | 56.8℃ | 52.4% |
| Pred_BP2984_BP2985 | F | tcattagcgcaagccgcagta | 21 | 59.9℃ | 52.4% |
| | R | ataggcggcctcgccagaggt | 21 | 65.5℃ | 66.7% |
| Pred_BP0170_BP0171 | F | ccatgcccgtgaaaacgggtt | 21 | 61.9℃ | 57.1% |
| | R | aaacccgttttcacgggcatg | 21 | 60.0℃ | 52.4% |
| Pred_BP3747_BP3748 | F | caagcatccgtctcatagggg | 21 | 58.6℃ | 57.1% |
| | R | acggatgcttgcggggcacct | 21 | 66.9℃ | 66.7% |
| Pred_BP3594_BP3595 | F | cctgtgctgcccccgcaacgg | 21 | 69.2℃ | 76.2% |
| | R | accgttgcgggggcagcacag | 21 | 68.1℃ | 71.4% |
| *bvgA* antisens (positive ctrl.) | R | cagcactcatgcccgtatcgt | 21 | 60.4℃ | 57.1% |

# Table S2: RNAz prediction details

| Genome coordinates (BAV-BP-BPP-BB)[1] | % Ident.[2] | SCI[3] | Zscore[4] | RNA proba.[5] | 5' gene | 5' gene function | 5' gene strand | 3' gene | 3' gene function | 3' gene strand |
|---|---|---|---|---|---|---|---|---|---|---|
| 9438..9588-10272..10422-10317..10467-10257..10407 | 85,2 | 0,91 | -2,19 | 0,975 | BP0011, *rplA* | 50S ribosomal protein L1 | > | BP0013, *rplJ* | 50S ribosomal protein L10 | > |
| 9448..9598-10282..10432-10327..10477-10267..10417 | 85,86 | 0,91 | -2,71 | 0,995 | | | | | | |
| 9458..9608-10292..10442-10337..10487-10277..10427 | 87,17 | 0,91 | -2,77 | 0,995 | | | | | | |
| 9468..9618-10302..10452-10347..10497-10287..10437 | 87,17 | 0,86 | -1,89 | 0,894 | | | | | | |
| 9478..9628-10312..10462-10357..10507-10297..10447 | 88,16 | 0,88 | -1,7 | 0,833 | | | | | | |
| 9488..9638-10322..10472-10367..10517-10307..10457 | 88,6 | 0,88 | -1,6 | 0,773 | | | | | | |
| 9498..9648-10332..10482-10377..10527-10317..10467 | 88,16 | 0,79 | -1,37 | 0,511 | | | | | | |
| 9508..9658-10342..10492-10387..10537-10327..10477 | 87,46 | 0,81 | -1,44 | 0,568 | | | | | | |
| 9528..9682-10362..10516-10407..10561-10347..10501 | 92,28 | 0,92 | -1,63 | 0,831 | | | | | | |
| 3629228..3629378-168917..169067-4661421..4661571-5223096..5223246 | 86,84 | 0,66 | -1,59 | 0,524 | BP0169 | hypothetical protein BP0169 | < | BP0170, *rhlE* | putative ATP-dependent RNA helicase | > |
| 500275..500425-197059..197209-817515..817665-901456..901606 | 89,11 | 0,79 | -1,41 | 0,532 | BP0194 | probable metal transporter | > | BP0195, *gcvT* | aminomethyltransferase | > |
| 500285..500435-197069..197219-817525..817675-901466..901616 | 89,44 | 0,83 | -1,5 | 0,636 | | | | | | |
| 500295..500445-197079..197229-817535..817685-901476..901626 | 89,55 | 0,84 | -1,86 | 0,861 | | | | | | |
| 500365..500515-197149..197299-817605..817755-901546..901696 | 84,29 | 0,73 | -1,77 | 0,741 | | | | | | |
| 3684103..3684253-481878..482028-4727820..4727970-5289495..5289645 | 89,25 | 0,96 | -3,01 | 0,999 | BP0470 | hypothetical protein BP0470 | > | BP 0471, *ribB* | 3,4-dihydroxy-2-butanone 4-phosphate synthase | > |
| 3684113..3684263-481888..482038-4727830..4727980-5289505..5289655 | 88,93 | 0,96 | -2,54 | 0,996 | | | | | | |
| 3684123..3684273-481898..482048-4727840..4727990-5289515..5289665 | 87,3 | 0,91 | -2,16 | 0,975 | | | | | | |
| 3684133..3684283-481908..482058-4727850..4728000-5289525..5289675 | 86,64 | 0,9 | -1,84 | 0,904 | | | | | | |
| 3684143..3684293-481918..482068-4727860..4728010-5289535..5289685 | 84,91 | 0,85 | -1,81 | 0,846 | | | | | | |
| 3684153..3684303-481928..482078-4727870..4728020-5289545..5289695 | 83,28 | 0,82 | -1,74 | 0,793 | | | | | | |
| 3684163..3684313-481938..482088-4727880..4728030-5289555..5289705 | 83,33 | 0,94 | -1,38 | 0,736 | | | | | | |
| 3684173..3684323-481948..482098-4727890..4728040-5289565..5289715 | 82,38 | 0,95 | -1,94 | 0,969 | | | | | | |
| 1187352..1187502-488504..488654-3573370..3573520-3989828..3989978 | 92,01 | 0,96 | -1,51 | 0,816 | BP0475 | ribonuclease E | < | BP0477 | ribosomal large subunit pseudouridine synthase C | > |
| 1112884..1113034-869031..869181-3658600..3658750-4073820..4073970 | 71,96 | 0,61 | -1,98 | 0,976 | BP0839 | transposase | < | BP0840 | outer membrane porin protein precursor | > |
| 1112894..1113044-869041..869191-3658610..3658760-4073830..4073980 | 72,8 | 0,63 | -2,04 | 0,983 | | | | | | |
| 1112904..1113054-869051..869201-3658620..3658770-4073840..4073990 | 72,6 | 0,62 | -2,12 | 0,989 | | | | | | |
| 1112914..1113064-869061..869211-3658630..3658780-4073850..4074000 | 73,41 | 0,64 | -2,63 | 0,999 | | | | | | |
| 1112924..1113078-869071..869225-3658640..3658794-4073860..4074014 | 74,93 | 0,63 | -2,18 | 0,981 | | | | | | |
| 1821216..1821293-1494130..1494207-1634573..1634650-2767844..2767921 | 90,38 | 1,03 | -1,13 | 0,627 | BP1418, *map* | methionine aminopeptidase | < | BP1419, *rpsB* | 30S ribosomal protein S2 | > |
| 1262010..1262105-2503141..2503236-3485762..3485857-3902190..3902285 | 71,86 | 0,68 | -1,61 | 0,912 | BP2364 | putative GntR-family transcriptional regulator | < | BP0477, *mdH* | malate dehydrogenase | > |
| 2922700..2922850-2624007..2624157-3746885..3747035-4160838..4160988 | 87,3 | 0,84 | -4,9 | 0,990 | BP2479 | putative integral membrane protein | < | BP2480, *kdpA* | potassium-transporting ATPase subunit A | > |
| 2922710..2922860-2624017..2624167-3746895..3747045-4160848..4160998 | 86,33 | 0,8 | -3,69 | 0,995 | | | | | | |
| 2922720..2922870-2624027..2624177-3746905..3747055-4160858..4161008 | 86,01 | 0,86 | -1,66 | 0,771 | | | | | | |
| 2922740..2922890-2624047..2624197-3746925..3747075-4160878..4161028 | 87,19 | 0,9 | -1,72 | 0,859 | | | | | | |
| 2922750..2922900-2624057..2624207-3746935..3747085-4160888..4161038 | 87,26 | 0,89 | -1,95 | 0,929 | | | | | | |
| 2922760..2922910-2624067..2624217-3746945..3747095-4160898..4161048 | 87,58 | 0,86 | -2,44 | 0,979 | | | | | | |
| 2922770..2922926-2624077..2624233-3746955..3747111-4160908..4161064 | 85,85 | 0,82 | -2,94 | 0,993 | | | | | | |
| 2491802..2491895-2699394..2699487-2831711..2831804-2221183..2221276 | 86,99 | 0,87 | -1,77 | 0,853 | BP2546, *recA* | recombinase A | < | BP2547 | two-component response regulatory protein | > |
| 2305333..2305483-3099570..3099720-2667432..2667582-2058071..2058221 | 90,16 | 0,86 | -3,51 | 0,994 | BP2908, *aroG* | 3-deoxy-7-phosphoheptulonate synthase | < | BP2909 | hypothetical protein | < |
| 2305343..2305493-3099580..3099730-2667442..2667592-2058081..2058231 | 89,84 | 0,89 | -3,12 | 0,996 | | | | | | |
| 2305353..2305503-3099590..3099740-2667452..2667602-2058091..2058241 | 90,05 | 0,88 | -3,43 | 0,996 | | | | | | |
| 3242911..3243061-3173584..3173734-4226978..4227128-4661736..4661886 | 88,56 | 0,82 | -3,87 | 0,992 | BP2982(p) | Pseudogene BP2982 | > | BP2983, *nrdA* | ribonucleotide-diphosphate reductase alpha subunit | |
| 3247376..3247526-3178090..3178240-4231484..4231634-4666242..4666392 | 89,07 | 0,89 | -4,48 | 0,994 | BP2984, nrdB | ribonucleotide-diphosphate reductase beta subunit | > | BP2985, *bpH1* | histone protein | > |
| 3247386..3247536-3178100..3178250-4231494..4231644-4666252..4666402 | 88,74 | 0,91 | -5,83 | 0,969 | | | | | | |
| 3247396..3247546-3178110..3178260-4231504..4231654-4666262..4666412 | 89,4 | 0,9 | -5,59 | 0,971 | | | | | | |
| 3247406..3247556-3178120..3178270-4231514..4231664-4666272..4666422 | 90,73 | 0,94 | -4,37 | 0,996 | | | | | | |
| 3247416..3247566-3178130..3178280-4231524..4231674-4666282..4666432 | 90,73 | 0,94 | -4,17 | 0,997 | | | | | | |
| 3247426..3247576-3178140..3178290-4231534..4231684-4666292..4666442 | 89,4 | 0,91 | -3,24 | 0,998 | | | | | | |
| 3247436..3247586-3178150..3178300-4231544..4231694-4666302..4666452 | 88,16 | 0,91 | -2,43 | 0,989 | | | | | | |
| 3247446..3247596-3178160..3178310-4231554..4231704-4666312..4666462 | 88,85 | 0,93 | -2,59 | 0,995 | | | | | | |
| 3247456..3247606-3178170..3178320-4231564..4231714-4666322..4666472 | 90,79 | 0,94 | -3,14 | 0,998 | | | | | | |
| 3247466..3247617-3178180..3178331-4231574..4231725-4666332..4666483 | 90,85 | 0,95 | -2,97 | 0,998 | | | | | | |
| 3157498..3157648-3246822..3246972-4086623..4086773-4493516..4493666 | 93,36 | 0,9 | -1,48 | 0,644 | BP3045(p) | Pseudogene BP3045 | < | BP3047 | hypothetical protein BP3047 | < |
| 2390216..2390302-3263729..3263815-213706..213792-210264..210350 | 82,26 | 0,8 | -1,61 | 0,672 | BP3061 | 5-formyltetrahydrofolate cyclo-ligase | > | BP3062 | putative integral membrane transport protein | > |
| 3486158..3486238-3605317..3605397-3836697..3836777-4234530..4234610 | 91,82 | 0,9 | -1,64 | 0,822 | BP3395 | putative ATP-dependent RNA helicase | < | BP3396 | putative conserved inner membrane protein | > |
| 2998739..2998874-3619230..3619365-3847380..3847515-4245358..4245493 | 88,45 | 0,84 | -2,25 | 0,954 | BP3410 | putative inner membrane protein | < | BP3411 | putative Xaa-Pro aminopeptidase | > |
| 2268865..2269015-3741851..3742001-2727966..2728116-2117293..2117443 | 81,46 | 0,81 | -3,32 | 0,999 | BP3529 | putative hydroxylase | > | BP3530, *hupB* | DNA-binding protein Hu-beta | > |
| 2268875..2269025-3741861..3742011-2727976..2728126-2117303..2117453 | 82,78 | 0,79 | -3,76 | 0,999 | | | | | | |
| 2268885..2269035-3741871..3742021-2727986..2728136-2117313..2117463 | 82,45 | 0,82 | -4,12 | 0,999 | | | | | | |
| 2268895..2269045-3741881..3742031-2727996..2728146-2117323..2117473 | 81,73 | 0,79 | -4,15 | 1,000 | | | | | | |
| 2268905..2269055-3741891..3742041-2728006..2728156-2117333..2117483 | 81,06 | 0,79 | -4,79 | 0,999 | | | | | | |
| 3340901..3341052-3811548..3811699-4317733..4317884-4752632..4752783 | 84,08 | 0,9 | -1,93 | 0,932 | BP3594, *metH* | 5-methyltetrahydrofolate--homocysteine methyltrans | < | BP3595 | putative outer membrane protein | > |
| 114591..114677-3896371..3896457-101817..101903-100620..100706 | 95,4 | 0,91 | -2,9 | 0,980 | BP3686 | conserved hypothetical protein | > | BP3687 | putative dihydrodipicolinate synthase | > |
| 3570899..3570996-3956837..3956934-4585612..4585709-5147268..5147365 | 91,46 | 0,91 | -3,79 | 0,996 | BP3747 | hypothetical protein BP3747 | > | BP3748, *rpoH* | RNA polymerase sigma factor | < |
| 3576618..3576688-3962134..3962204-4590910..4590980-5152566..5152636 | 85,21 | 0,78 | -1,69 | 0,725 | BP3752 | hypothetical protein BP3752 | > | BP3753, *gltB* | glutamate synthase [NADPH] large chain precursor | > |
| 3726354..3726504-4077015..4077165-4764310..4764460-5329938..5330088 | 83,33 | 0,8 | -2,16 | 0,943 | BP3864 | hypothetical protein BP3864 | < | BP3865 | putative response regulator protein | > |
| 3726364..3726514-4077025..4077175-4764320..4764470-5329948..5330098 | 83 | 0,79 | -2,22 | 0,952 | | | | | | |
| 3726374..3726524-4077035..4077185-4764330..4764480-5329958..5330108 | 83,67 | 0,69 | -1,94 | 0,796 | | | | | | |

1- Coordinates of predictions on B. Avium (BAV), B. pertussis (BP), B. parapertussis (BPP), B. bronchiseptica (BB). 2- Observed percentage identity during Blast Analysis
3- Structure Conservation Index as calculated by RNAz. 4- Z-score as calculated by RNAz. 5- P-value as calculated by RNAz.

# Table S4: BRE details

| Acc.number | Strain | % identity[1] | Alignment length | Mismat.[2] | Gap openings | Query start | Query end | Subject start | Subject end | Orientation | E-value | Bit score | Repeat localisation[3] | 5' Gene | 5' Gene Symbol | 5' gene strand | 3' Gene | 3' Gene Symbol | 3' Gene strand | Within gene | Within gene strand |
|---|---|---|---|---|---|---|---|---|---|---|---|---|---|---|---|---|---|---|---|---|---|
| NC_008060.1 | Burkholderia cenocepacia AU 1054 chromosome 1 | 70.83 | 72 | 19 | 2 | 9 | 78 | 858348 | 858277 | - | 0.022 | 39.6 | IGR | Bcen0790 | | + | Bcen0791 | | + | | |
| NC_008062.1 | Burkholderia cenocepacia AU 1054 chromosome 3 | 69.62 | 79 | 12 | 12 | 12 | 90 | 674795 | 674865 | + | 0.052 | 38.3 | IGR | Bcen6075 | | + | Bcen6076 | | + | | |
| NC_008391.1 | Burkholderia cepacia AMMD chromosome 2 | 82 | 50 | 7 | 2 | 11 | 58 | 1E+06 | 1436251 | + | 0.00019 | 46.5 | IGR | Bamb 4489 | | - | Bamb 4490 | | - | | |
| NC_008542.1 | Burkholderia cenocepacia HI2424 chromosome 1 | 70.83 | 72 | 19 | 2 | 9 | 78 | 1E+06 | 1402912 | - | 0.016 | 40.1 | IGR | Bcen2424 1271 | | + | Bcen2424 1272 | | + | | |
| NC_008542.1 | Burkholderia cenocepacia HI2424 chromosome 1 | 65.52 | 87 | 21 | 9 | 1 | 78 | 2E+06 | 2216841 | - | 0.025 | 39.4 | IGR | Bcen2424 2001 | | - | Bcen2424 2002 | | - | | |
| NC_008687.1 | Paracoccus denitrificans PD1222 chromosome 2 | 67.69 | 65 | 20 | 1 | 27 | 90 | 1E+06 | 1358295 | - | 0.082 | 37.8 | IGR | Pden 4199 | | - | Pden 4200 | | + | | |
| NC_008702.1 | Azoarcus sp. BH72 | 80 | 45 | 7 | 2 | 8 | 50 | 721017 | 721061 | + | 0.066 | 38.1 | IGR | azo0683 | *ytfL1* | + | azo0684 | *mdcY* | - | | |
| NC_008702.1 | Azoarcus sp. BH72 | 75.47 | 53 | 11 | 2 | 8 | 58 | 721165 | 721217 | + | 0.035 | 39 | IGR | azo0683 | *ytfL1* | + | azo0684 | *mdcY* | - | | |
| NC_008702.1 | Azoarcus sp. BH72 | 75.38 | 65 | 9 | 7 | 8 | 72 | 721315 | 721376 | + | 0.00049 | 45.2 | IGR | azo0683 | *ytfL1* | + | azo0684 | *mdcY* | - | | |
| NC_008752.1 | Acidovorax avenae subsp. citrulli AAC00-1 | 77.03 | 74 | 14 | 3 | 10 | 83 | 202919 | 202991 | + | 1.20E-06 | 53.8 | Gene | | | | | | | Aave0181 | + |
| NC_008752.1 | Acidovorax avenae subsp. citrulli AAC00-1 | 71.08 | 83 | 16 | 8 | 5 | 85 | 2E+06 | 2221253 | + | 0.012 | 40.5 | Gene | | | | | | | Aave2039 | + |
| NC_008752.1 | Acidovorax avenae subsp. citrulli AAC00-1 | 75 | 76 | 15 | 4 | 10 | 85 | 2E+06 | 2221337 | + | 2.50E-05 | 49.5 | Gene | | | | | | | Aave2039 | + |
| NC_008752.1 | Acidovorax avenae subsp. citrulli AAC00-1 | 76.32 | 76 | 14 | 4 | 10 | 85 | 2E+06 | 2221421 | + | 4.50E-06 | 51.9 | Gene | | | | | | | Aave2039 | + |
| NC_008752.1 | Acidovorax avenae subsp. citrulli AAC00-1 | 76.32 | 76 | 14 | 4 | 10 | 85 | 2E+06 | 2221505 | + | 4.50E-06 | 51.9 | Gene | | | | | | | Aave2039 | + |
| NC_008752.1 | Acidovorax avenae subsp. citrulli AAC00-1 | 76.32 | 76 | 14 | 4 | 10 | 85 | 2E+06 | 2221589 | + | 4.50E-06 | 51.9 | Gene | | | | | | | Aave2039 | + |
| NC_008752.1 | Acidovorax avenae subsp. citrulli AAC00-1 | 75 | 76 | 15 | 4 | 12 | 87 | 3E+06 | 3474958 | + | 0.0004 | 45.5 | Gene | | | | | | | Aave3147 | + |
| NC_008782.1 | Acidovorax sp. JS42 | 75.34 | 73 | 13 | 5 | 12 | 83 | 59097 | 59169 | + | 0.00049 | 45.2 | Gene | | | | | | | Ajs0054 | + |
| NC_008782.1 | Acidovorax sp. JS42 | 72.22 | 90 | 21 | 4 | 1 | 90 | 4E+06 | 4339298 | + | 3.30E-08 | 59 | Overlap | Ajs4068 | *trmU* | - | Ajs4069 | | + | | |
| NC_008781.1 | Polaromonas naphthalenivorans CJ2 | 76.19 | 84 | 17 | 3 | 7 | 90 | 141273 | 141355 | + | 7.50E-09 | 61.2 | IGR | Pnap0128 | | - | Pnap0129 | | - | | |
| NC_008781.1 | Polaromonas naphthalenivorans CJ2 | 72.97 | 74 | 15 | 5 | 13 | 86 | 159347 | 159279 | - | 0.0027 | 42.7 | Overlap | Pnap0148 | | + | Pnap0149 | | + | | |
| NC_008781.1 | Polaromonas naphthalenivorans CJ2 | 73.97 | 73 | 12 | 7 | 13 | 85 | 178752 | 178819 | + | 0.0033 | 42.4 | IGR | | | | | | | Pnap0167 | - |
| NC_008781.1 | Polaromonas naphthalenivorans CJ2 | 73.42 | 79 | 15 | 6 | 12 | 90 | 187153 | 187079 | - | 0.00049 | 45.2 | Overlap | Pnap0175 | | + | Pnap0176 | | + | | |
| NC_008781.1 | Polaromonas naphthalenivorans CJ2 | 71.01 | 69 | 17 | 3 | 13 | 81 | 806129 | 806196 | + | 0.066 | 38.1 | Gene | | | | | | | Pnap0760 | - |
| NC_008781.1 | Polaromonas naphthalenivorans CJ2 | 72.22 | 72 | 17 | 3 | 12 | 83 | 807767 | 807837 | + | 0.0063 | 41.5 | Gene | | | | | | | Pnap0763 | - |
| NC_008781.1 | Polaromonas naphthalenivorans CJ2 | 74.36 | 78 | 17 | 3 | 12 | 89 | 2E+06 | 2070933 | - | 2.00E-05 | 49.8 | Gene | | | | | | | Pnap1948 | + |
| NC_008781.1 | Polaromonas naphthalenivorans CJ2 | 72.15 | 79 | 19 | 3 | 12 | 90 | 2E+06 | 2344989 | + | 0.00014 | 47 | IGR | Pnap2196 | *rho* | + | Pnap2197 | | + | | |
| NC_008781.1 | Polaromonas naphthalenivorans CJ2 | 72.73 | 77 | 14 | 7 | 13 | 89 | 3E+06 | 2726829 | + | 0.0033 | 42.4 | Gene | | | | | | | Pnap2592 | + |
| NC_008781.1 | Polaromonas naphthalenivorans CJ2 | 73.08 | 78 | 13 | 8 | 12 | 89 | 3E+06 | 2852089 | + | 0.015 | 40.2 | IGR | Pnap2704 | | - | Pnap2705 | | - | | |
| NC_008781.1 | Polaromonas naphthalenivorans CJ2 | 75.64 | 78 | 18 | 1 | 12 | 89 | 3E+06 | 2915230 | + | 2.30E-07 | 56.2 | Gene | | | | | | | Pnap2771 | + |
| NC_008781.1 | Polaromonas naphthalenivorans CJ2 | 75 | 72 | 14 | 4 | 12 | 83 | 3E+06 | 2925235 | - | 0.0033 | 42.4 | Gene | | | | | | | Pnap2781 | - |
| NC_008781.1 | Polaromonas naphthalenivorans CJ2 | 71.23 | 73 | 16 | 5 | 13 | 85 | 3E+06 | 3456712 | + | 0.028 | 39.3 | Gene | | | | | | | Pnap3255 | - |
| NC_008781.1 | Polaromonas naphthalenivorans CJ2 | 75 | 80 | 19 | 1 | 10 | 89 | 3E+06 | 3456800 | + | 2.30E-07 | 56.2 | Gene | | | | | | | Pnap3255 | - |
| NC_008781.1 | Polaromonas naphthalenivorans CJ2 | 71.43 | 77 | 17 | 5 | 13 | 89 | 4E+06 | 3596554 | + | 0.0014 | 43.6 | IGR | Pnap3390 | | - | Pnap3391 | | - | | |
| NC_008781.1 | Polaromonas naphthalenivorans CJ2 | 75.64 | 78 | 16 | 3 | 12 | 89 | 4E+06 | 3786055 | + | 2.40E-06 | 52.9 | Gene | | | | | | | Pnap3565 | + |
| NC_008786.1 | Verminephrobacter eiseniae EF01-2 | 81.82 | 55 | 8 | 2 | 6 | 58 | 1E+06 | 1415677 | + | 5.80E-05 | 48.2 | Gene | | | | | | | Veis1279 | + |
| NC_008786.1 | Verminephrobacter eiseniae EF01-2 | 83.64 | 55 | 7 | 2 | 6 | 58 | 1E+06 | 1415755 | + | 8.50E-06 | 51 | Gene | | | | | | | Veis1279 | + |
| NC_008825.1 | Methylibium petroleiphilum PM1 | 70.83 | 72 | 18 | 3 | 12 | 83 | 1E+06 | 1478811 | + | 0.012 | 40.5 | Gene | | | | | | | Mpe_A1374 | + |
| NC_008825.1 | Methylibium petroleiphilum PM1 | 73.68 | 76 | 12 | 8 | 12 | 87 | 2E+06 | 1540925 | - | 5.80E-05 | 48.2 | Gene | | | | | | | Mpe_A1431 | - |
| NC_009445.1 | Bradyrhizobium sp. ORS278 | 75.47 | 53 | 13 | 0 | 26 | 78 | 525087 | 525035 | - | 0.016 | 40 | Overlap | BRADO0518 | | + | BRADO0519 | | + | | |
| NC_009445.1 | Bradyrhizobium sp. ORS278 | 72.55 | 51 | 14 | 0 | 26 | 76 | 6E+06 | 6211422 | + | 0.087 | 37.5 | Gene | | | | | | | BRADo5979 | - |
| NC_010002.1 | Delftia acidovorans SPH-1 | 78.26 | 69 | 12 | 3 | 6 | 71 | 66057 | 65989 | - | 2.70E-06 | 52.6 | IGR | Daci 0060 | | + | Dac 061 | | - | | |
| NC_010002.1 | Delftia acidovorans SPH-1 | 83.02 | 53 | 8 | 1 | 3 | 54 | 624049 | 624101 | + | 4.20E-06 | 51.9 | IGR | Daci0567 | | + | Daci 0568 | | + | | |
| NC_010002.1 | Delftia acidovorans SPH-1 | 73.33 | 90 | 20 | 4 | 4 | 89 | 646023 | 645934 | - | 1.30E-08 | 60.2 | IGR | Daci0585 | | - | Daci 0586 | | - | | |
| NC_010002.1 | Delftia acidovorans SPH-1 | 73.91 | 92 | 19 | 5 | 4 | 90 | 980861 | 980770 | - | 6.70E-09 | 61.2 | IGR | Daci 0873 | | - | Daci 0874 | | - | | |
| NC_010002.1 | Delftia acidovorans SPH-1 | 74.73 | 91 | 20 | 3 | 3 | 90 | 2E+06 | 1566184 | + | 3.00E-10 | 65.7 | IGR | Daci 1407 | | + | Daci 1408 | | + | | |
| NC_010002.1 | Delftia acidovorans SPH-1 | 72.37 | 76 | 19 | 2 | 4 | 77 | 2E+06 | 1860517 | - | 3.10E-05 | 49 | IGR | Daci 1666 | | - | Daci 1667 | | + | | |
| NC_010002.1 | Delftia acidovorans SPH-1 | 74.44 | 90 | 21 | 2 | 3 | 90 | 2E+06 | 1862140 | - | 2.20E-09 | 62.8 | IGR | Daci 1668 | | + | Daci 1669 | | - | | |
| NC_010002.1 | Delftia acidovorans SPH-1 | 76.92 | 91 | 18 | 3 | 3 | 90 | 2E+06 | 2005240 | - | 3.50E-12 | 72.1 | IGR | Daci 1807 | | - | Daci 1808 | | - | | |
| NC_010002.1 | Delftia acidovorans SPH-1 | 70.33 | 91 | 23 | 4 | 4 | 90 | 2E+06 | 2013859 | - | 8.80E-07 | 54.2 | IGR | Daci 1817 | | + | Daci 1818 | | - | | |
| NC_010002.1 | Delftia acidovorans SPH-1 | 69.23 | 91 | 22 | 6 | 1 | 89 | 2E+06 | 2462023 | + | 1.30E-05 | 50.3 | IGR | Daci 2242 | | + | Daci 2243 | | + | | |
| NC_010002.1 | Delftia acidovorans SPH-1 | 80 | 90 | 15 | 3 | 4 | 90 | 2E+06 | 2463332 | + | 2.80E-12 | 72.4 | IGR | Daci 2243 | | + | Daci 2244 | | + | | |
| NC_010002.1 | Delftia acidovorans SPH-1 | 80 | 90 | 15 | 3 | 4 | 90 | 3E+06 | 3440692 | + | 2.40E-13 | 75.9 | Gene | | | | | | | Daci 3122 | - |
| NC_010002.1 | Delftia acidovorans SPH-1 | 70.97 | 93 | 23 | 4 | 2 | 90 | 4E+06 | 4100423 | - | 3.60E-07 | 55.4 | Overlap | Daci 3731 | | + | Daci 3732 | | + | | |
| NC_010002.1 | Delftia acidovorans SPH-1 | 79.12 | 91 | 16 | 3 | 3 | 90 | 5E+06 | 5310064 | + | 2.00E-13 | 76.3 | IGR | Daci 4810 | | - | Daci 4811 | | + | | |
| NC_010002.1 | Delftia acidovorans SPH-1 | 76.92 | 52 | 11 | 1 | 3 | 54 | 7E+06 | 6567616 | - | 0.004 | 42 | IGR | Daci 5904 | | + | Daci 5905 | | - | | |
| NC_010002.1 | Delftia acidovorans SPH-1 | 68.42 | 95 | 21 | 9 | 3 | 90 | 7E+06 | 6624112 | + | 0.037 | 38.8 | IGR | Daci5942 | | + | Daci 5943 | | + | | |
| NC_003888.3 | Streptomyces coelicolor A3(2) | 77.78 | 45 | 10 | 0 | 39 | 83 | 2E+06 | 1534288 | + | 0.055 | 38.6 | IGR | SC01436 | | - | SC01437 | - | | | |
| NC_006513.1 | Azoarcus sp. EbN1 | 76.6 | 47 | 9 | 2 | 9 | 53 | 593159 | 593205 | + | 0.04 | 38.4 | IGR | ebA1013 | | + | ebA1017 | | + | | |
| NC_006513.1 | Azoarcus sp. EbN1 | 75.64 | 78 | 13 | 6 | 9 | 84 | 2E+06 | 2110043 | + | 4.50E-06 | 51.5 | Overlap | ebA3567 | *ribAB* | - | ebA3569 | *ribE* | - | | |
| NC_006513.1 | Azoarcus sp. EbN1 | 67.09 | 79 | 15 | 11 | 11 | 89 | 2E+06 | 2182865 | + | 0.025 | 39.1 | IGR | ebA 3703 | | - | ebA 3705 | | - | | |
| NC_006513.1 | Azoarcus sp. EbN1 | 78.26 | 46 | 8 | 2 | 7 | 50 | 2E+06 | 2470185 | + | 0.025 | 39.1 | Gene | | | | | | | ebA 4187 | hyfG |
| NC_006513.1 | Azoarcus sp. EbN1 | 68.97 | 87 | 17 | 10 | 3 | 89 | 4E+06 | 3697279 | + | 0.00054 | 44.6 | Overlap | ebA6242 | | - | ebA6243 | | - | | |
| NC_006513.1 | Azoarcus sp. EbN1 | 67.14 | 70 | 21 | 2 | 11 | 78 | 4E+06 | 3927080 | - | 0.082 | 37.4 | IGR | ebA6624 | *trbC* | + | ebA6625 | *traN* | + | | |
| NC_007298.1 | Dechloromonas aromatica RCB | 70.13 | 77 | 13 | 10 | 11 | 87 | 4E+06 | 4283325 | + | 0.0054 | 41.6 | IGR | Daro 3987 | | - | Daro 3988 | | - | | |
| NC_007508.1 | Xanthomonas campestris pv. vesicatoria str. 85-10 | 72.73 | 55 | 15 | 0 | 25 | 79 | 889642 | 889696 | + | 0.049 | 38.4 | Gene | | | | | | | XCV0776 | betT |
| NC_007510.1 | Burkholderia sp. 383 chromosome 1 | 68.57 | 70 | 20 | 2 | 11 | 78 | 742635 | 742566 | - | 0.096 | 37.4 | IGR | Bcep18194 A3835 | *hemH* | + | Bcep18194 A3836 | | + | | |
| NC_007908.1 | Rhodoferax ferrireducens T118 | 71.05 | 76 | 18 | 4 | 11 | 86 | 147060 | 147135 | + | 7.00E-05 | 47.9 | IGR | Rfer 0143 | | - | Rfer0144 | | - | | |
| NC_007908.1 | Rhodoferax ferrireducens T118 | 70.59 | 85 | 21 | 4 | 6 | 90 | 2E+06 | 2309790 | - | 8.60E-05 | 47.6 | Gene | | | | | | | Rfer 2131 | + |
| NC_007908.1 | Rhodoferax ferrireducens T118 | 77.38 | 84 | 16 | 3 | 7 | 90 | 3E+06 | 3327005 | + | 1.70E-08 | 59.9 | IGR | Rfer 3016 | | - | Rfer 3017 | | - | | |
| NC_007908.1 | Rhodoferax ferrireducens T118 | 74.7 | 83 | 17 | 4 | 8 | 90 | 4E+06 | 3580129 | + | 9.90E-07 | 54 | Gene | | | | | | | Rfer 3231 | - |
| NC_007908.1 | Rhodoferax ferrireducens T118 | 79.01 | 81 | 14 | 3 | 10 | 90 | 4E+06 | 3812202 | + | 5.20E-07 | 54.9 | Gene | | | | | | | Rfer 3425 | - |
| NC_007908.1 | Rhodoferax ferrireducens T118 | 80.82 | 73 | 9 | 5 | 12 | 83 | 4E+06 | 4431292 | + | 4.20E-07 | 55.3 | Gene | | | | | | | Rfer 3963 | + |
| NC_007908.1 | Rhodoferax ferrireducens T118 | 72.37 | 76 | 19 | 2 | 12 | 87 | 5E+06 | 4513308 | + | 5.60E-05 | 48.2 | Gene | | | | | | | Rfer 4048 & Rfer 4049 | |
| NC_007908.1 | Rhodoferax ferrireducens T118 | 69.74 | 76 | 19 | 4 | 9 | 84 | 5E+06 | 4531341 | - | 0.00073 | 44.5 | IGR | Rfer 4063 | | + | Rfer 4064 | | + | | |
| NC_007948.1 | Polaromonas sp. JS666 | 83.33 | 48 | 8 | 0 | 7 | 54 | 79433 | 79480 | + | 0.00011 | 47.3 | IGR | Bpro 0075 | | + | Bpro 0076 | | + | | |
| NC_007948.1 | Polaromonas sp. JS666 | 71.62 | 74 | 15 | 6 | 12 | 85 | 186517 | 186448 | - | 0.098 | 37.4 | Overlap | Bpro 0171 | | - | Bpro 0172 | | + | | |
| NC_007948.1 | Polaromonas sp. JS666 | 72.97 | 74 | 18 | 2 | 12 | 85 | 216227 | 216300 | + | 0.00038 | 45.4 | IGR | Bpro 0197 | | - | Bpro 0198 | | - | | |
| NC_007948.1 | Polaromonas sp. JS666 | 71.62 | 74 | 20 | 1 | 12 | 85 | 236500 | 236572 | + | 0.0017 | 43.3 | Gene | | | | | | | Bpro 0216 | - |
| NC_007948.1 | Polaromonas sp. JS666 | 69.74 | 76 | 22 | 1 | 12 | 87 | 236581 | 236655 | + | 0.0061 | 41.4 | Gene | | | | | | | Bpro 0216 | - |
| NC_007948.1 | Polaromonas sp. JS666 | 67.95 | 78 | 20 | 5 | 13 | 90 | 245061 | 245133 | + | 0.098 | 37.4 | IGR | Bpro 0224 | | + | Bpro 0225 | | + | | |
| NC_007948.1 | Polaromonas sp. JS666 | 80 | 50 | 6 | 4 | 3 | 52 | 261213 | 261260 | + | 0.0026 | 42.7 | IGR | Bpro 0237 | | - | Bpro 0238 | | - | | |
| NC_007948.1 | Polaromonas sp. JS666 | 76.39 | 72 | 14 | 3 | 12 | 83 | 264699 | 264769 | + | 4.60E-05 | 48.5 | Gene | | | | | | | Bpro 0242 | + |
| NC_007948.1 | Polaromonas sp. JS666 | 69.62 | 79 | 20 | 4 | 7 | 85 | 304215 | 304139 | - | 0.064 | 38 | IGR | Bpro 0288 | | + | Bpro 0289 | | - | | |
| NC_007948.1 | Polaromonas sp. JS666 | 69.62 | 79 | 23 | 1 | 12 | 90 | 355242 | 355165 | - | 0.0014 | 43.6 | Gene | | | | | | | Bpro 0334 | + |
| NC_007948.1 | Polaromonas sp. JS666 | 71.43 | 77 | 16 | 6 | 13 | 89 | 410375 | 410447 | + | 0.012 | 40.5 | Overlap | Bpro 0397 | | + | Bpro 0398 | | - | | |
| NC_007948.1 | Polaromonas sp. JS666 | 72.37 | 76 | 15 | 6 | 12 | 87 | 461084 | 461155 | + | 0.0076 | 41.1 | Overlap | Bpro 0442 | | + | Bpro 0443 | | - | | |
| NC_007948.1 | Polaromonas sp. JS666 | 77.03 | 74 | 15 | 2 | 10 | 83 | 577858 | 577931 | + | 6.70E-06 | 51.3 | Gene | | | | | | | Bpro 0575 | - |
| NC_007948.1 | Polaromonas sp. JS666 | 72.31 | 65 | 14 | 4 | 4 | 68 | 604257 | 604319 | + | 0.014 | 40.2 | IGR | Bpro 0598 | | - | Bpro 0599 | | - | | |
| NC_007948.1 | Polaromonas sp. JS666 | 70.51 | 78 | 17 | 6 | 13 | 90 | 676403 | 676474 | + | 0.064 | 38 | Overlap | Bpro 0663 | | + | Bpro 0664 | | - | | |
| NC_007948.1 | Polaromonas sp. JS666 | 69.62 | 79 | 18 | 6 | 12 | 90 | 834777 | 834851 | + | 0.079 | 37.7 | Overlap | Bpro 0808 | | + | Bpro 0809 | | + | | |
| NC_007948.1 | Polaromonas sp. JS666 | 75.64 | 78 | 16 | 3 | 7 | 83 | 876614 | 876691 | + | 5.40E-06 | 51.6 | IGR | Bpro 0855 | | - | Bpro 0856 | | - | | |
| NC_007948.1 | Polaromonas sp. JS666 | 73.08 | 78 | 15 | 6 | 13 | 90 | 890932 | 890859 | - | 0.0021 | 43 | Overlap | Bpro 0866 | *aspS* | + | Bpro 0867 | *ntpA* | + | | |
| NC_007948.1 | Polaromonas sp. JS666 | 84.62 | 52 | 7 | 1 | 4 | 54 | 977295 | 977346 | + | 0.027 | 39.3 | IGR | Bpro 0953 | | + | Bpro 0954 | | - | | |
| NC_007948.1 | Polaromonas sp. JS666 | 78.43 | 51 | 11 | 0 | 4 | 54 | 977379 | 977429 | + | 0.0009 | 44.2 | IGR | Bpro 0953 | | + | Bpro 0954 | | - | | |
| NC_007948.1 | Polaromonas sp. JS666 | 75.31 | 81 | 16 | 4 | 10 | 90 | 1E+06 | 1029249 | - | 2.90E-06 | 52.5 | Gene | | | | | | | Bpro 1012 | - |
| NC_007948.1 | Polaromonas sp. JS666 | 75.95 | 79 | 18 | 1 | 7 | 85 | 1E+06 | 1030423 | - | 7.60E-08 | 57.7 | Gene | | | | | | | Bpro 1013 | - |
| NC_007948.1 | Polaromonas sp. JS666 | 75 | 80 | 15 | 5 | 7 | 86 | 1E+06 | 1084441 | - | 0.00048 | 45.1 | IGR | Bpro 1056 | | + | Bpro 1057 | | - | | |
| NC_007948.1 | Polaromonas sp. JS666 | 75.9 | 83 | 16 | 4 | 7 | 89 | 1E+06 | 1090992 | - | 4.20E-07 | 55.3 | Gene | | | | | | | Bpro 1062 | + |
| NC_007948.1 | Polaromonas sp. JS666 | 72 | 75 | 16 | 5 | 9 | 83 | 1E+06 | 1107816 | + | 0.042 | 38.7 | Gene | | | | | | | Bpro 1075 | + |
| NC_007948.1 | Polaromonas sp. JS666 | 75 | 72 | 16 | 2 | 12 | 83 | 1E+06 | 1134546 | + | 0.00013 | 47 | IGR | Bpro 1100 | | + | Bpro 1101 | | + | | |
| NC_007948.1 | Polaromonas sp. JS666 | 69.62 | 79 | 20 | 4 | 12 | 90 | 1E+06 | 1188242 | - | 0.004 | 42 | Overlap | Bpro 1145 | *pyr B* | + | Bpro 1146 | | + | | |
| NC_007948.1 | Polaromonas sp. JS666 | 71.23 | 73 | 18 | 3 | 13 | 85 | 1E+06 | 1188331 | - | 0.018 | 39.9 | Overlap | Bpro 1145 | *pyr B* | + | Bpro 1146 | | + | | |
| NC_007948.1 | Polaromonas sp. JS666 | 89.8 | 49 | 4 | 1 | 6 | 54 | 1E+06 | 1268951 | + | 3.50E-06 | 52.2 | gene | Bpro 1219 | | - | Bpro 1220 | | - | | |
| NC_007948.1 | Polaromonas sp. JS666 | 82.35 | 51 | 7 | 2 | 37 | 87 | 1E+06 | 1297104 | - | 0.0061 | 41.4 | IGR | Bpro 1252 | | + | Bpro 1253 | | + | | |
| NC_007948.1 | Polaromonas sp. JS666 | 69.23 | 78 | 19 | 5 | 13 | 90 | 1E+06 | 1373229 | + | 0.018 | 39.9 | IGR | Bpro 1332 | | + | Bpro 1333 | | - | | |
| NC_007948.1 | Polaromonas sp. JS666 | 76.19 | 84 | 16 | 4 | 4 | 87 | 2E+06 | 1516752 | + | 1.80E-07 | 56.5 | Gene | | | | | | | Bpro 1465 | - |
| NC_007948.1 | Polaromonas sp. JS666 | 73.17 | 82 | 21 | 1 | 7 | 88 | 2E+06 | 1641770 | - | 5.20E-07 | 54.9 | Gene | | | | | | | Bpro 1589 | - |
| NC_007948.1 | Polaromonas sp. JS666 | 75.68 | 74 | 15 | 3 | 12 | 85 | 2E+06 | 1656093 | + | 5.40E-06 | 51.6 | Gene | | | | | | | Bpro 1604 | + |
| NC_007948.1 | Polaromonas sp. JS666 | 77.03 | 74 | 14 | 3 | 12 | 85 | 2E+06 | 1656177 | + | 2.30E-06 | 52.8 | Gene | | | | | | | Bpro 1604 | + |
| NC_007948.1 | Polaromonas sp. JS666 | 75.64 | 78 | 16 | 3 | 12 | 89 | 2E+06 | 1670972 | + | 1.00E-05 | 50.6 | IGR | Bpro 1618 | | - | Bpro 1619 | | - | | |
| NC_007948.1 | Polaromonas sp. JS666 | 76.62 | 77 | 15 | 3 | 7 | 83 | 2E+06 | 1741878 | + | 6.40E-07 | 54.6 | Overlap | Bpro 1690 | | + | Bpro 1691 | | + | | |
| NC_007948.1 | Polaromonas sp. JS666 | 77.11 | 83 | 18 | 1 | 7 | 89 | 2E+06 | 1926431 | - | 4.60E-10 | 65.1 | Gene | | | | | | | Bpro 1866 | - |
| NC_007948.1 | Polaromonas sp. JS666 | 74.03 | 77 | 17 | 3 | 12 | 88 | 2E+06 | 1928909 | + | 1.90E-05 | 49.7 | Overlap | Bpro 1866 | | - | Bpro 1867 | | - | | |
| NC_007948.1 | Polaromonas sp. JS666 | 71.08 | 83 | 23 | 1 | 7 | 89 | 2E+06 | 2180179 | - | 6.70E-06 | 51.3 | IGR | Bpro 2092 | | + | Bpro 2093 | | + | | |
| NC_007948.1 | Polaromonas sp. JS666 | 72.86 | 70 | 13 | 6 | 13 | 82 | 2E+06 | 2218666 | + | 0.042 | 38.7 | IGR | Bpro 2126 | | - | Bpro 2127 | | - | | |
| NC_007948.1 | Polaromonas sp. JS666 | 71.79 | 78 | 16 | 6 | 13 | 90 | 2E+06 | 2218878 | + | 0.0026 | 42.7 | IGR | Bpro 2126 | | - | Bpro 2127 | | - | | |
| NC_007948.1 | Polaromonas sp. JS666 | 77.38 | 84 | 18 | 1 | 7 | 90 | 2E+06 | 2228154 | - | 1.30E-09 | 63.6 | IGR | Bpro 2136 | | + | Bpro 2137 | | + | | |
| NC_007948.1 | Polaromonas sp. JS666 | 74.68 | 79 | 14 | 6 | 12 | 90 | 2E+06 | 2262280 | - | 0.00016 | 46.6 | Overlap | Bpro 2167 | | + | Bpro 2168 | | + | | |
| NC_007948.1 | Polaromonas sp. JS666 | 72.15 | 79 | 19 | 3 | 12 | 90 | 2E+06 | 2291869 | - | 0.0002 | 46.3 | Gene | | | | | | | Bpro 2196 | + |
| NC_007948.1 | Polaromonas sp. JS666 | 72.73 | 77 | 13 | 8 | 13 | 89 | 2E+06 | 2381872 | + | 0.022 | 39.6 | IGR | Bpro 2288 | *moaA* | - | Bpro 2289 | | - | | |
| NC_007948.1 | Polaromonas sp. JS666 | 75.68 | 74 | 14 | 4 | 12 | 85 | 3E+06 | 2543983 | - | 0.00031 | 45.7 | IGR | Bpro 2440 | | + | Bpo 2441 | *guaA* | + | | |
| NC_007948.1 | Polaromonas sp. JS666 | 69.14 | 81 | 22 | 3 | 11 | 90 | 3E+06 | 2696042 | + | 0.00031 | 45.7 | IGR | Bpro 2574 | | - | Bpro 2575 | | - | | |
| NC_007948.1 | Polaromonas sp. JS666 | 87.5 | 48 | 5 | 1 | 7 | 54 | 3E+06 | 2726467 | + | 3.70E-05 | 48.8 | IGR | Bpro 2595 | | - | Bpro 2596 | | - | | |
| NC_007948.1 | Polaromonas sp. JS666 | 70.11 | 87 | 22 | 4 | 6 | 90 | 3E+06 | 2847389 | - | 0.0021 | 43 | IGR | Bpro 2702 | | - | Bpro 2703 | | - | | |
| NC_007948.1 | Polaromonas sp. JS666 | 67.95 | 78 | 24 | 1 | 12 | 89 | 3E+06 | 2904379 | + | 0.042 | 38.7 | Gene | | | | | | | Bpro 2760 | - |
| NC_007948.1 | Polaromonas sp. JS666 | 88.68 | 53 | 5 | 1 | 3 | 54 | 3E+06 | 2951700 | - | 3.40E-07 | 55.6 | Gene | | | | | | | Bpro 2807 | - |
| NC_007948.1 | Polaromonas sp. JS666 | 73.08 | 78 | 18 | 3 | 12 | 89 | 3E+06 | 3057914 | + | 3.00E-05 | 49.1 | Overlap | Bpro 2895 | | + | Bpro 2896 | | - | | |
| NC_007948.1 | Polaromonas sp. JS666 | 73.33 | 75 | 17 | 3 | 16 | 90 | 3E+06 | 3110162 | - | 0.0002 | 46.3 | Overlap | Bpro 2947 | | + | Bpro 2948 | | + | | |
| NC_007948.1 | Polaromonas sp. JS666 | 71.95 | 82 | 20 | 3 | 3 | 84 | 3E+06 | 3265076 | + | 3.70E-05 | 48.8 | IGR | Bpro 3095 | | - | Bpro 3096 | | + | | |
| NC_007948.1 | Polaromonas sp. JS666 | 72.5 | 80 | 17 | 5 | 12 | 90 | 3E+06 | 3332019 | - | 0.004 | 42 | IGR | Bpro 3155 | | - | Bpro 3156 | | - | | |
| NC_007948.1 | Polaromonas sp. JS666 | 73.24 | 71 | 15 | 4 | 13 | 83 | 3E+06 | 3332111 | - | 0.034 | 39 | IGR | Bpro 3155 | | - | Bpro 3156 | | - | | |
| NC_007948.1 | Polaromonas sp. JS666 | 69.62 | 79 | 19 | 5 | 12 | 90 | 3E+06 | 3364709 | - | 0.034 | 39 | IGR | Bpro 3180 | | - | Bpro 3181 | | + | | |
| NC_007948.1 | Polaromonas sp. JS666 | 90.48 | 42 | 4 | 0 | 7 | 48 | 3E+06 | 3398301 | + | 3.00E-05 | 49.1 | IGR | Bpro 3211 | | + | Bpro 3212 | | + | | |
| NC_007948.1 | Polaromonas sp. JS666 | 75.34 | 73 | 12 | 6 | 13 | 85 | 3E+06 | 3455710 | + | 0.0076 | 41.1 | Overlap | Bpro 3264 | | + | Bpro 3265 | | - | | |
| NC_007948.1 | Polaromonas sp. JS666 | 72.09 | 86 | 19 | 5 | 6 | 88 | 3E+06 | 3481130 | + | 3.00E-05 | 49.1 | IGR | Bpro 3283 | | + | | *rpsU* | + | | |
| NC_007948.1 | Polaromonas sp. JS666 | 83.33 | 42 | 7 | 0 | 7 | 48 | 4E+06 | 3678198 | + | 0.0061 | 41.4 | IGR | Bpro 3474 | | + | Bpro 3475 | | + | | |
| NC_007948.1 | Polaromonas sp. JS666 | 72.97 | 74 | 18 | 2 | 12 | 85 | 4E+06 | 3754065 | + | 0.00048 | 45.1 | IGR | Bpro 3553 | | + | Bpro 3254 | | + | | |
| NC_007948.1 | Polaromonas sp. JS666 | 80.95 | 84 | 14 | 2 | 7 | 90 | 4E+06 | 3754154 | + | 1.50E-11 | 70 | IGR | Bpro 3553 | | + | Bpro 3254 | | + | | |
| NC_007948.1 | Polaromonas sp. JS666 | 82.43 | 74 | 10 | 3 | 10 | 83 | 4E+06 | 3787898 | - | 1.80E-07 | 56.5 | Gene | | | | | | | Bpro 3589 | + |
| NC_007948.1 | Polaromonas sp. JS666 | 77.03 | 74 | 14 | 3 | 12 | 85 | 4E+06 | 3800019 | - | 1.00E-05 | 50.6 | Gene | | | | | | | Bpro 3597 | - |
| NC_007948.1 | Polaromonas sp. JS666 | 77.22 | 79 | 15 | 3 | 7 | 85 | 4E+06 | 3820329 | + | 1.80E-07 | 56.5 | Gene | | | | | | | Bpro 3616 | + |
| NC_007948.1 | Polaromonas sp. JS666 | 70.89 | 79 | 16 | 7 | 12 | 90 | 4E+06 | 3835571 | + | 0.018 | 39.9 | Overlap | Bpro 3633 | | - | Bpro 3634 | | - | | |
| NC_007948.1 | Polaromonas sp. JS666 | 74.32 | 74 | 14 | 5 | 12 | 85 | 4E+06 | 3864044 | - | 0.004 | 42 | Gene | | | | | | | Bpro 3662 | - |
| NC_007948.1 | Polaromonas sp. JS666 | 77.11 | 83 | 15 | 4 | 6 | 88 | 4E+06 | 3872992 | - | 9.50E-08 | 57.4 | Gene | | | | | | | Bpro 3667 | + |
| NC_007948.1 | Polaromonas sp. JS666 | 72.86 | 70 | 16 | 3 | 16 | 85 | 4E+06 | 3873079 | - | 0.018 | 39.9 | Gene | | | | | | | Bpro 3667 | + |
| NC_007948.1 | Polaromonas sp. JS666 | 88.37 | 43 | 5 | 0 | 7 | 49 | 4E+06 | 4054193 | + | 4.60E-05 | 48.5 | IGR | Bpro 3862 | | - | Bpro 3863 | | - | | |
| NC_007948.1 | Polaromonas sp. JS666 | 78.21 | 78 | 12 | 5 | 12 | 89 | 4E+06 | 4085288 | - | 8.60E-05 | 47.6 | Gene | | | | | | | Bpro 3892 | + |
| NC_007948.1 | Polaromonas sp. JS666 | 76.62 | 77 | 17 | 1 | 7 | 83 | 4E+06 | 4242421 | - | 9.50E-08 | 57.4 | IGR | Bpro 4041 | | - | Bpro 4042 | | + | | |
| NC_007948.1 | Polaromonas sp. JS666 | 75.32 | 77 | 15 | 4 | 12 | 88 | 4E+06 | 4289245 | - | 0.00038 | 45.4 | IGR | Bpro 4078 | | + | Bpro 4079 | | + | | |
| NC_007948.1 | Polaromonas sp. JS666 | 75.9 | 83 | 19 | 1 | 7 | 89 | 4E+06 | 4369057 | - | 3.90E-09 | 62 | IGR | Bpro 4154 | | - | Bpro 4155 | | - | | |
| NC_007948.1 | Polaromonas sp. JS666 | 78.38 | 74 | 13 | 3 | 10 | 83 | 4E+06 | 4406301 | + | 3.50E-06 | 52.2 | Gene | | | | | | | Bpro 4193 | + |
| NC_007948.1 | Polaromonas sp. JS666 | 75 | 68 | 14 | 3 | 16 | 83 | 4E+06 | 4469368 | - | 0.0021 | 43 | Gene | | | | | | | Bpro 4242 | + |
| NC_007948.1 | Polaromonas sp. JS666 | 82.5 | 80 | 12 | 2 | 7 | 86 | 4E+06 | 4498006 | + | 3.60E-11 | 68.8 | Gene | | | | | | | Bpro 4265 | - |
| NC_007948.1 | Polaromonas sp. JS666 | 77.03 | 74 | 14 | 3 | 10 | 83 | 5E+06 | 4504276 | + | 1.50E-06 | 53.4 | Gene | | | | | | | Bpro 4272 | + |
| NC_007948.1 | Polaromonas sp. JS666 | 74.07 | 81 | 20 | 1 | 10 | 90 | 5E+06 | 4710415 | - | 6.40E-07 | 54.6 | Gene | | | | | | | Bpro 4457 | - |
| NC_007948.1 | Polaromonas sp. JS666 | 73.08 | 78 | 15 | 6 | 13 | 90 | 5E+06 | 4739321 | + | 0.0009 | 44.2 | IGR | Bpro4484 | | - | Bpro 4485 | | - | | |
| NC_007948.1 | Polaromonas sp. JS666 | 69.44 | 72 | 21 | 1 | 12 | 83 | 5E+06 | 4811768 | - | 0.022 | 39.6 | Gene | | | | | | | Bpro 4548 | + |
| NC_007948.1 | Polaromonas sp. JS666 | 77.78 | 81 | 15 | 3 | 7 | 87 | 5E+06 | 4832720 | + | 1.40E-08 | 60.2 | Gene | | | | | | | Bpro 4567 | - |
| NC_007948.1 | Polaromonas sp. JS666 | 74.68 | 79 | 14 | 6 | 12 | 90 | 5E+06 | 4945509 | - | 0.0011 | 43.9 | Gene | | | | | | | Bpro 4656 | + |
| NC_007948.1 | Polaromonas sp. JS666 | 67.57 | 74 | 23 | 1 | 16 | 89 | 5E+06 | 4947663 | - | 0.064 | 38 | Gene | | | | | | | Bpro 4656 | + |
| NC_007948.1 | Polaromonas sp. JS666 | 72.37 | 76 | 15 | 6 | 12 | 87 | 5E+06 | 4947749 | - | 0.004 | 42 | Gene | | | | | | | Bpro 4656 | + |
| NC_007948.1 | Polaromonas sp. JS666 | 77.78 | 54 | 10 | 2 | 37 | 90 | 5E+06 | 4975532 | + | 0.042 | 38.7 | Overlap | Bpro 4687 | | + | Bpro 4688 | | + | | |
| NC_007948.1 | Polaromonas sp. JS666 | 70.51 | 78 | 16 | 7 | 13 | 90 | 5E+06 | 4993488 | - | 0.034 | 39 | IGR | Bpro 4702 | | + | Bpro 4703 | | + | | |
| NC_007948.1 | Polaromonas sp. JS666 | 71.88 | 64 | 16 | 2 | 27 | 90 | 5E+06 | 5006955 | - | 0.042 | 38.7 | Overlap | Bpro 4717 | | + | Bpro 4718 | | - | | |
| NC_007948.1 | Polaromonas sp. JS666 | 73.44 | 64 | 16 | 1 | 27 | 90 | 5E+06 | 5007058 | - | 0.0026 | 42.7 | IGR | Bpro 4717 | | + | Bpro 4718 | | - | | |
| NC_007948.1 | Polaromonas sp. JS666 | 73.44 | 64 | 16 | 1 | 27 | 90 | 5E+06 | 5007160 | - | 0.0026 | 42.7 | IGR | Bpro 4717 | | + | Bpro 4718 | | - | | |
| NC_007948.1 | Polaromonas sp. JS666 | 80.49 | 82 | 13 | 3 | 6 | 87 | 5E+06 | 5152852 | - | 2.00E-10 | 66.3 | Gene | | | | | | | Bpro 4864 | + |
| NC_007948.1 | Polaromonas sp. JS666 | 73.61 | 72 | 16 | 3 | 12 | 83 | 5E+06 | 5154899 | + | 0.00031 | 45.7 | Gene | | | | | | | Bpro 4866 | + |
| NC_007948.1 | Polaromonas sp. JS666 | 70.89 | 79 | 18 | 5 | 12 | 90 | 5E+06 | 5174534 | - | 0.018 | 39.9 | Gene | | | | | | | Bpro 4885 | - |
| NC_007964.1 | Nitrobacter hamburgensis X14 | 67.09 | 79 | 25 | 1 | 9 | 87 | 4E+06 | 4109484 | + | 0.064 | 38 | Gene | | | | | | | Bpro 3914 | + |

1- Observed percentage identity during YASS Analysis. 2- Number of mismatches. 3- BRE localization: IGR = within intergenic region, Gene = within gene ORF, Overlap = overlapping IGR and gene ORF. Bit Score < 50 are underligned in gray. Multi-repeat locus are designated by a square

# List S1 of Bordetelle BRE sequences and positions

>STRAIN NAME [genomic coordinates]orientation
Sequence
>BORDETELLA PERTUSSIS_[2208359-2208448]+
TTTTTTTTGCCGCCGGGCCGCCCCAAGGCAAAAAAGCCCCCTCGGGGGGCAGCAAGCCCGCACAGCGGGCGCAGCGTGGGGGTCTTTTT
>BORDETELLA PERTUSSIS_[1164089-1164175]+
TTTTTTTGCCGCCGGGCCGCCCCAAGGCAAAAAAGCCCCCTTGGGGGGCAGCAAGCCCGCACAGCGGGCGCAGCGTGGGGGCCTTTT
>BORDETELLA PERTUSSIS_[1164003-1164093]+
TTTTTTTTTTGCCGCCGGGCCGCCCCAAGGCAAAAAAACCCCCTTGGGGGGCAGCAAGCCCGCACAGCGGGCGCAGCGTGGGGGCCTTTTT
>BORDETELLA PERTUSSIS_[2703634-2703724]+
TTTTTTTTTTGCCGCCGGGCCGCCCCAAGGCAAAAAAGCCTCCTCGGGGGGCAGCAAGCCCGCATAGCGGGCGCAGCGTGGGGGCCTTTTT
>BORDETELLA PERTUSSIS_[1883961-1884042]+
TTTTTTTTGCCGCCGGGCCGCCCCAAGGCAAAAAAGCCCCCTCGGGGGGCAGCAAGCCCGCATAGCGGGCGCAGCGTGGGGG
>BORDETELLA PERTUSSIS_[1096455-1096543]+
TTTTTTTTTTGCCGCCGGGCCGCCCCAAGGCAAAAAGCCCCCTCGGGGGGCAGCAAGCCCGCCCAGCGGGCGCAGCGTGGGGGGGGTTT
>BORDETELLA PERTUSSIS_[1884049-1884131]+
TTTGCCGCTGGGCCGCCCCAAGGCAAAAAGCCCCCTCGGGGGGCAGCAAGCCCGCGTAGCGGGCGCAGCGCGGGGGCTTCTTT
>BORDETELLA PERTUSSIS_[3605317-3605401]+
TTTTTTGCCGCCGGGCCGCCCCAAGGCAAAAGCCCCCCTGGGGGGCAGCAAGCTGGCGCAGCGAGCGCAGCGTGGGGGCTTTTCT
>BORDETELLA BRONCHISEPTICA_[1460738-1460827]+
TTTTTTTTTGCCGCCGGGACGCCCCAAGGCAAAAAAGCCCCCTCGGGGGGCAGCAAGCCCGCACAGCGGGCGCAGCGTGGGGGCCTTTTT
>BORDETELLA BRONCHISEPTICA_[2434758-2434845]+
TTTTTTTGCCGCCGGGCCGCCCCAAGGCAAAAAAGCCCCCTTGGGGGGCAGCAAGCCCGCACAGCGGGCGCAGCGTGGGGGCCTTTTT
>BORDETELLA BRONCHISEPTICA_[2434841-2434928]+
TTTTTTTGCCGCCGGGCCGCCCCAAGGCAAAAAAGCCCCCTTGGGGGGCAGCAAGCCCGCACAGCGGGCGCAGCGTGGGGGCCTTTTT
>BORDETELLA BRONCHISEPTICA_[2434924-2435011]+
TTTTTTTGCCGCCGGGCCGCCCCAAGGCAAAAAAGCCCCCTTGGGGGGCAGCAAGCCCGCACAGCGGGCGCAGCGTGGGGGCCTTTTT
>BORDETELLA BRONCHISEPTICA_[2435007-2435094]+
TTTTTTTGCCGCCGGGCCGCCCCAAGGCAAAAAAGCCCCCTTGGGGGGCAGCAAGCTCGCACAGCGGGCGCAGCGTGGGGGCCTTTTT
>BORDETELLA BRONCHISEPTICA_[2434588-2434678]+
TTTTTTTTTTGCCGCCGGGCCGCCCCAAGGCAAAAAAGCCCCCTTGGGGGGCAGCAAGCCCGCATAGCGGGCGCAGCGTGGGGGCCTTTTT
>BORDETELLA BRONCHISEPTICA_[2435174-2435260]+
TTTTTTTGCCGCCGGGCCGCCCCAAGGCAAAAAAGCCCCCTTTGGGGGCAGCAAGCCCGCACAGCGGGCGCAGCGTGGGGGCCTTTT
>BORDETELLA BRONCHISEPTICA_[2434674-2434762]+
TTTTTTTGCCGCCGGGCCGCCCCAAGGCAAAAAAGCCCCCTTGGGGGGGCAGCAAGCCCGCACAGCGGGCGCAGCGTGGGGGCCTTTTT
>BORDETELLA BRONCHISEPTICA_[2435090-2435178]+
TTTTTTTGCCGCCGGGCCGCCCCAAGGCAAAAAAGCCCCCTTGGGGGGGCAGCAAGCCCGCACAGCGGGCGCAGCGTGGGGGCCTTTTT
>BORDETELLA BRONCHISEPTICA_[2216947-2217037]-
TTTTTTTTTGCCGCCGGGCCGCCCCAAGGCAAAAAAGCCCCCTCGGGGGGCAGCAAGCCCGCATAGCGGGCGCAGCGTGGGGGCCTTTTTT
>BORDETELLA BRONCHISEPTICA_[3565086-3565166]-
TTTTTTTGCCGCCGGGCCGCCCCAAGGCAAAAAAGCCCCCTCGGGGGGCAGCAAGCCCGCACAGCGGGCGCAGCGTGGGGG
>BORDETELLA BRONCHISEPTICA_[1460823-1460903]+
TTTTTTTGCCGCCGGGCCGCCCCAAGGCAAAAAAGCCCCCTCGGGGGGCAGCAAGCCCGCACAGCGGGCGCAGCGTGGGGG
>BORDETELLA BRONCHISEPTICA_[1460911-1460994]+
TTTGCCGCCGGGCCGCTCCAAGGCAAAAAAGCCCCCTCGGGGGGCAGCAAGCCCGCACAGCGGGCGCAGCGTGGGGGCCTTTTT
>BORDETELLA BRONCHISEPTICA_[1505406-1505493]-
TTTTTTGCCGCCGGGCCGCCCCAAGGCAAAAAAGCCCCCTTGGGGGGCAGCAAGCCCGCACAGCGGGCGCAGCGTGGGGGCCTTTTTT
>BORDETELLA BRONCHISEPTICA_[2401245-2401326]+
TTTTTTTTGCCGCCGGGCCGCCCCAAGGCAAAAAAGCCCCCTCGGGGGGCAGCAAGCCCGCATAGCGGGCGCAGCGTGGGGG
>BORDETELLA BRONCHISEPTICA_[2401416-2401499]+
TTTGCCGCCGGGCCGCCCCAAGGCAAAAAAGCCCCCTCGGGGGGCAGCAAGCCCGCATAGCAGGCGCAGCGTGGGGGCTTCTTT
>BORDETELLA BRONCHISEPTICA_[2237660-2237747]+
TTTTTTTGCCGCCGGGCCGTCCCAAGGCAAAAAGGCCCCCTTGGGGGGCAGCAAGCGCGCGCAGCGGGCGCAGCGTGGGGGCATTCTT
>BORDETELLA BRONCHISEPTICA_[2237576-2237664]+
TTTTTTTTGCCGCCGGGCCGTCCCAAGGCAAAAAGGCCCCCTTGGGGGGCAGCAAGCGCGCGCAGCGGGCGCAGCGTGGGGGCGTTTTT
>BORDETELLA BRONCHISEPTICA_[2401333-2401409]+
TTTGCCGCCGGGCCGCCCCAAGGCAAAAAAGCCCCCTCGGGGGGCAGCAAGCCCGCATAGCGGGCGCAGCGTGGGGG
>BORDETELLA BRONCHISEPTICA_[4234530-4234613]+
TTTTTTGCCGCCGGGCCGCCCCAAGGCAAAAGCCCCCCTGGGGGGCAGCAAGCTCGCGCAGCGAGCGCAGCGTGGGGGCATTTT
>BORDETELLA BRONCHISEPTICA_[4234610-4234694]+
TTTTCCACCGCCGGGCCGCCCCAAGGCAAAAGCCCCCCTGGGGGGCAGCAAGCTGGCGCAGCGAACGCAGCGTGGGGGCTTTTCT
>BORDETELLA BRONCHISEPTICA_[3565027-3565078]-
TTTGCCGCCGGGCCGCCCCAAGGCAAAAAAGCCCCCTCGGGGGGCAAAGCGA
>BORDETELLA PARAPERTUSSIS_[1228628-1228710]+
TTTTTTTTTGCCGCCGGGCCGCTCCAAGGCAAAAAAGCCCCCTCGGGGGGCAGCAAGCCCGCACAGCGGGCGCAGCGTGGGGG
>BORDETELLA PARAPERTUSSIS_[2038933-2039020]+
TTTTTTTGCCGCCGGGCCGTCCCAAGGCAAAAAGGCCCCCTTGGGGGGCAGCAAGCCCGCGCAGCGGGCGCAGCGTGGGGGCGTTTTT
>BORDETELLA PARAPERTUSSIS_[2039016-2039103]+
TTTTTTTGCCGCCGGGCCGTCCCAAGGCAAAAAGGCCCCCTTGGGGGGCAGCAAGCCCGCGCAGCGGGCGCAGCGTGGGGGCGTTTTT
>BORDETELLA PARAPERTUSSIS_[2039099-2039186]+
TTTTTTTGCCGCCGAGCCGTCCCAAGGCAAAAAGGCCCCCTTGGGGGGCAGCAAGCCCGCGCAGCGGGCGCAGCGTGGGGGCATTCTT
>BORDETELLA PARAPERTUSSIS_[2163265-2163348]+
TTTGCCGCCGGGCCGCCCCAAGGCAAAAAAGCCCCCTCGGGGGGCAGCAAGCCCGCATAGCGGGCGCAGCGTGGGGCACTACTT
>BORDETELLA PARAPERTUSSIS_[2163347-2163425]+
TTTGCCGCCGGGCCGCCCCAAGGCAAAAAAGCCCCCTCGGGGGGCAGCAAGCCCGCATAGCGGGCGCAGCATGGGGGCC
>BORDETELLA PARAPERTUSSIS_[2163182-2163258]+
TTTGCCGCCGGGCCGCCCCAAGGCAAAAAGGCCCCCTCGGGGGGCAGCAAGCCCGCGTAGCGGGCGCAGCGTGGGGG
>BORDETELLA PARAPERTUSSIS_[2163094-2163175]+
TTTTTTTT-GCCGCCGGGCCGTCCCAAGGCAGAAAGGCCCCCTTGGGGGGCAGCAAGCCCGCGTAGCGGGCGCAGCGTGGGGG

>BORDETELLA PARAPERTUSSIS_[3836697-3836781]+
TTTTTTGCCGCCGGGCCGCCCCAAGGCAAAAGCCCCC--CTGGGGGGCAGCAAGCTGGCGCAACGAGCGCAGCGTGGGGGCTTTTCT
>BORDETELLA PARAPERTUSSIS_[1897463-1897512]+
TTTTTTTGCCGCCGGGCCGCCCCAAGGCAAAAAAGCCCCCTCGGGGGGCA
>BORDETELLA AVIUM_[2150764-2150853]+
TTTGCCCCGCCGCCGGGCCGCCCCAAGGCGGGTAGCGCCCCCTCGGGGGGCAGCAAGCGCGCCAAGCGCGCGCAGCGTGGGGGTTTTTTT
>BORDETELLA AVIUM_[3486158-3486236]+
TTTTTTATCGCCGGGCCGTCCCAAGATAAAAAGCCCCCTTGGGGGGCAGCAAGCTCGCACAGCGGGCGCAGCGTGGGGG
>BORDETELLA AVIUM_[3455352-3455426]+
GCCGACGGGCCGTCCCTAGGCGGGACAGCCCCCTCGGGGGGCAGCAAGCAGGCGATAGCCTGCGCAGCGTGGGGG
>BORDETELLA AVIUM_[3455184-3455259]+
GCCGACGGGCCGCCCCTAGGCGGGACAGCCCCCTCGGGGGGCAGCAAGCAGGCGACAAGCCTGCGCAGCGTGGGGG
>BORDETELLA AVIUM_[3486248-3486316]+
CGCCGGACCGTCCCAAGATAGAGCCCCCTTGGGGGGCAGCAAGCTCGCACAGCGGGCGCAGCGTGGGGG
>BORDETELLA AVIUM_[720273-720336]+
TTTTTTATCGTAGGGCCGCCCCAAGATAGCCCCCTCGGGGGGCAGCAAGCGCGCGAAGCGTGCG
>BORDETELLA AVIUM_[3455268-3455343]+
GCCGACGGGCCGCCCCTAGGCGGGACAGCCCCCTCAGAGAACAGCAAGCAAGCGACAAGCCTGCGCAGCGTGGGGG
>BORDETELLA AVIUM_[2150681-2150766]+
TTGCCCCGCCGCCGAGCCGTCCCGAGGCGGGCAGCGCCCCCCTCGGGGGGCAGCAAGCGCGCCAAGCGCGCGCAGCGTGGGGGTTT
