# Supplementary material for: Detection of small RNAs in Bordetella pertussis and identification of a novel repeated genetic element
Source: BMC Genomics. 2011 Apr 27;12:207. doi: 10.1186/1471-2164-12-207 (PMC3110155; doi:10.1186/1471-2164-12-207)
Supplement: Additional file 2 — Additional figure S2 - Additional table S3. Figure S2: Test for independent transcription between the putative sRNA positions and the upstream or downstream ORF and RT-PCR primers. Table S3: List of primers used in PCR described in figure S2. [file 1471-2164-12-207-S2.PDF]

Supplementary figure S2

Putative sRNA upstream of the coding gene

Putative sRNA downstream of the coding gene

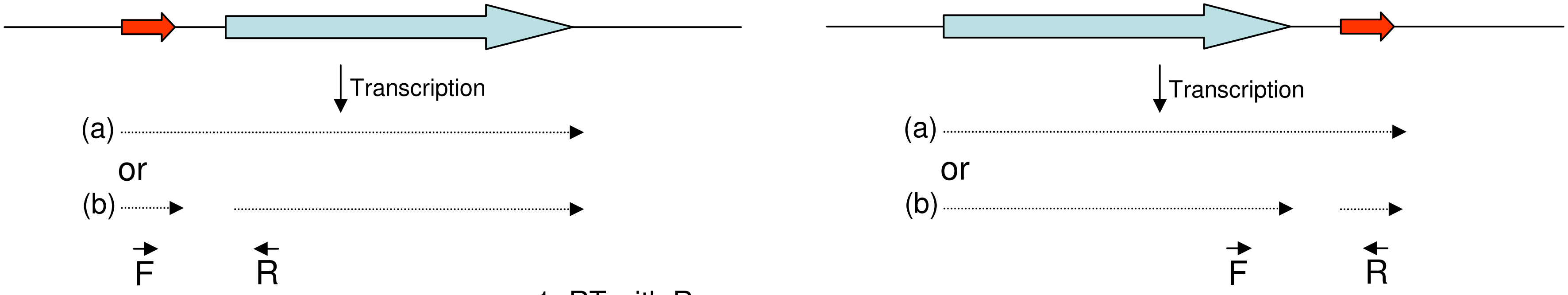

1- RT with R
2- PCR with F+P

Case (a): single transcription unit => PCR +
Case (b): independent transcription units => PCR -

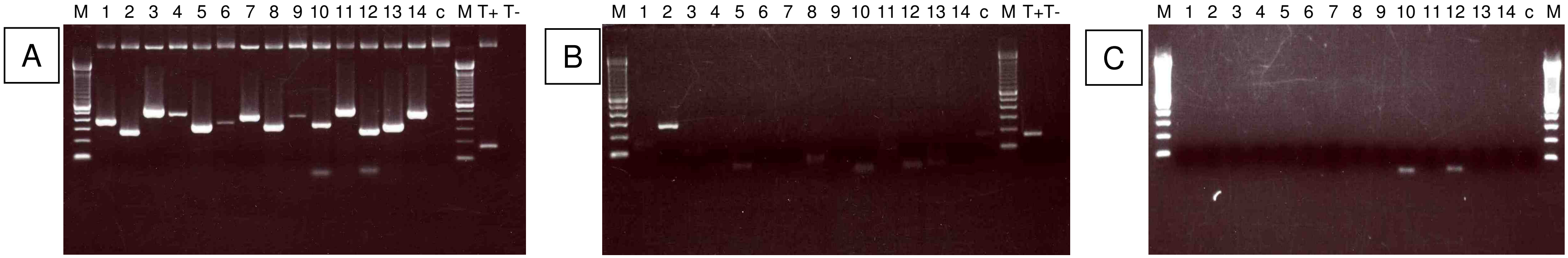

Figure S1: Test for independent transcription between the putative sRNA positions and the upstream or downstream ORF
Primer oligonucleotides were designed as described on the schema depending on the upstream or downstream position of the putative sRNA relative to the neighbored ORF. The RT was then initiated using primer in position R followed by a PCR using a pair of primers in positions F and R. A single transcription unit (cases (a)) should result in a positive PCR (detection of a band at the expected size) and independent transcription units (cases (b)) should results in a negative PCR (no band). A- The primer pairs (see list in Supplementary Table 3) were tested on genomic DNA to check PCR efficiency and obtained sizes. B- RT-PCRs were performed on extracted RNA as described. C- Control RT-PCRs were performed in absence of RT enzyme to test for DNA contamination in RNA template.
Lanes 1 to 14: see supplementary table 3 below. Lane c: PCR control without primers. Lane T+: RT-PCR positive control on *bvgA* 5'UTR.
Lane T-: PCR and RT-PCR negative control with no template in reaction.

Table S3

Lane nbr. on the gel	Small transcript names	Position of putative sRNA relative to adjacent ORF	Adjacent ORF	Primer type (F or R)	Sequence (5'-3')
1	TranscriptA1 & A2	Upstream	BP0475	F	tacagtccgcagcccgagtga
				R	tggacgatcagctcctggc
2	TranscriptB	Upstream	BP1419	F	tagcgcaagacttaaccctcgg
				R	cttcggttgccaccagttcg
3	TranscriptC	Upstream	BP1879	F	gcatgagtgctgctaaac
				R	gttactaacggaagacccg
4	TranscriptD1 & D2	Upstream	BP2479	F	gtcccatgcaggtgaggag
				R	cggttcatgaccagcgca
5	TranscriptE1 & E2	Upstream	BP2547	F	catcccctgtgccagaatggcc
				R	caggcccttgacgcgctgttc
6	TranscriptF1 & F2	Upstream	BP2908	F	aagggacgcgtgttaact
				R	gcttgagacgatccgcgta
7	TranscriptF1 & F2	Downstream	BP2909	F	ttaccaacggcaagttcg
				R	agttaacacgcgtccctt
8	TranscriptH	Upstream	BP2985	F	acctctggcgaggccgcctat
				R	cgcagctttcttggccggagtg
9	TranscriptH	Downstream	BP2984	F	gacgccggagttccgcga
				R	ataggcggcctcgccagaggt
10	TranscriptK	Upstream	BP3410	F	cgcgatgcgttcaccctg
				R	cataggccagcgtcgccag
11	TranscriptL1 & L2	Upstream	BP3594	F	gttgcgggggcagcacaggctg
				R	gatcgacgtggcgccgaaggtg
12	TranscriptL'	Upstream	BP3595	F	ctgtgctgcccccgcaacggtc
				R	gatcgcgcggcggtgacgac
13	TranscriptM'	Upstream	BP3687	F	gtcagatgggcgacggaacagc
				R	tcctccagcacggcgtcgag
14	TranscriptM'	Downstream	BP3686	F	gaccgttccagggcaagc
				R	ggaacagcaagctccggtcaga
T+	5'UTR (control)	Upstream	*bvgA* (BP1878)	F	ccgtatcgttgcttgctgacg
				R	atcggtttcgccgatgact
